# Blood flow restriction added to usual care exercise in patients with early weight bearing restrictions after cartilage or meniscus repair in the knee joint: a feasibility study

**DOI:** 10.1186/s40634-022-00533-4

**Published:** 2022-10-04

**Authors:** Thomas Linding Jakobsen, Kristian Thorborg, Jakob Fisker, Thomas Kallemose, Thomas Bandholm

**Affiliations:** 1 Centre of Rehabilitation, City of Copenhagen, Copenhagen, Denmark; 2 Physical Medicine & Rehabilitation Research – Copenhagen (PMR-C), Department of Physical and Occupational Therapy, Amager and Hvidovre Hospital, Hvidovre, Denmark; 3 Department of Clinical Medicine, University of Copenhagen, Copenhagen, Denmark; 4 Department of Clinical Research, Amager and Hvidovre Hospital, Hvidovre, Denmark; 5 Department of Orthopedic Surgery, Amager and Hvidovre Hospital, Hvidovre, Denmark

**Keywords:** Blood flow restriction exercise, Meniscus, Cartilage, Surgery, Rehabilitation, Feasibility studies

## Abstract

**Purpose:**

Blood flow restriction – low load strength training (BFR-LLST) is theoretically superior to traditional heavy strength training when rehabilitating patients who cannot heavily load tissues following surgery. The main purpose of this study was to examine the feasibility of BFR-LLST added to usual care exercise early after cartilage or meniscus repair in the knee joint.

**Methods:**

We included 42 patients with cartilage (*n* = 21) or meniscus repair (*n* = 21) of the knee joint. They attended 9 weeks of BFR-LLST added to a usual care exercise program at an outpatient rehabilitation center. Outcome measures were assessed at different time points from four (baseline) to 26 weeks postoperatively and included adherence, harms, knee joint and thigh pain, perceived exertion, thigh circumference (muscle size proxy), isometric knee-extension strength, self-reported disability and quality of life.

**Results:**

On average, patients with cartilage or meniscus repair completed > 84% of the total BFR-LLST supervised sessions. Thirty-eight patients reported 146 adverse events of which none were considered serious. No decrease in thigh circumference or exacerbation of knee joint or quadriceps muscle pain of the operated leg was found in either group during the intervention period.

**Conclusions:**

BFR-LLST added to usual care exercise initiated early after cartilage or meniscus repair seems feasible and may prevent disuse thigh muscle atrophy during a period of weight bearing restrictions. Harms were reported, but no serious adverse events were found. Our findings are promising but need replication using a RCT-design.

**Trial registration:**

NCT03371901, preprint (open access): https://www.medrxiv.org/content/10.1101/2022.03.31.22272398v1

**Supplementary Information:**

The online version contains supplementary material available at 10.1186/s40634-022-00533-4.

## Background

Cartilage or meniscus repairs in the knee joint are common orthopedic procedures, in which patients early after surgery are restricted from full weight bearing in the full range of motion and high impact activities of the knee joint. The consequence of reduced weightbearing and arthrogenic inhibition of the quadriceps muscle following knee surgery is a pronounced and persistent decrease of knee-extension strength in the operated leg [25, 41, 66, 76, 82]. The loss of muscle strength may delay return to normal daily activities (e.g., stair climbing), work/sports activities and negatively affect quality of life. An exercise modality to increase muscle strength is moderate blood flow restriction during low-load strength training (BFR-LLST) - also called occlusion training [48, 73]. BFR-LLST involves application of a wrapping device such as an inflated tourniquet/cuff or an elastic band [48] to partially restrict the arterial inflow and venous outflow to muscle(s) during exercise. The amount of blood flow to the muscles distal to the cuff is preferably controlled with an individualized pressure [62] determined using for example, Doppler ultrasound [40], a hand-held oximeter [9, 85] or a set applied pressure [15]. BFR-LLST (20–40% of 1-Repetition Maximum (RM)) requires much less external loading than traditional strength training (70–80% of 1RM). BFR-LLST produces positive training adaptations, such as muscle hypertrophy and increased strength in the lower extremity in healthy subjects, and patients with knee pathology [27, 47, 73, 79]. The underlying mechanisms are not fully understood, but may stem from a complex interplay of reduction in oxygen delivery to the muscle (hypoxia), accumulation of metabolites, muscle fiber recruitment and proliferation of myogenic stem cells [56, 63, 69]. Few studies have shown increased muscle strength and self-reported physical function and quality of life in patients who followed a rehabilitation program with BFR-LLST added after knee surgery [24, 29, 59, 78, 79].

To our knowledge, early BFR-LLST added to usual care exercise has never been investigated in patients recovering from cartilage or meniscus repair in the knee joint, although some recommend BFR-LLST clinically to encourage the return of quadriceps function and facilitate an earlier return to sport [70]. In clinical practice, acute knee joint and quadriceps muscle pain, perceptual responses, and fear of adverse events are potentially limiting factors for the application and effect of BFR-LLST [12, 28, 54, 74].

The main aim of this study was to investigate the feasibility of BFR-LLST added to usual care exercise on adherence, adverse events, knee-related symptoms and muscle mass early after cartilage or meniscus repair. Additionally, clinical outcomes were assessed to describe changes over time when patients with cartilage or meniscus repair followed a program of BFR-LLST added to usual care exercise.

## Material and methods

**Fig. 1:**
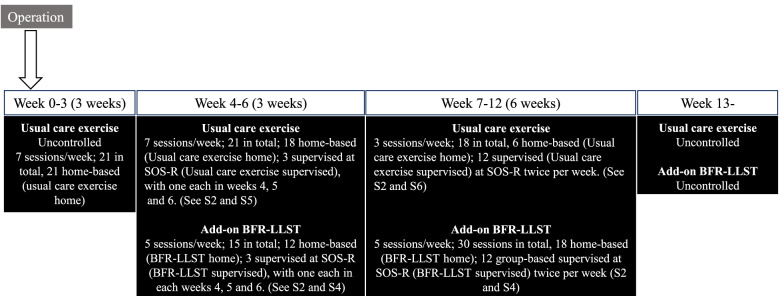
Overview of study treatment, testing and assessment timeline. Uncontrolled; Treatment provided prior to study inclusion or after the intervention period. SOS-R; Section for Orthopedic and sports Rehabilitation. BFR-LLST; Blood flow restriction – low load strength training

This exploratory prospective study used consecutive sampling to assess the feasibility of 9 weeks of BFR-LLST added to a usual care exercise program at a rehabilitation center, Section for Orthopedic and Sports Rehabilitation (SOS-R), Nørrebro, City of Copenhagen. To enhance early recovery, the BFR-LLST knee-extension exercise was added to the usual care exercise program [11]. Patients performed BFR-LLST knee-extension without external load for the first 6 weeks and subsequently for 6 weeks with external load. When loaded BFR-LLST knee extensions were introduced at 6 weeks, they replaced the usual care knee-extensions that were part of the usual care exercise program. The study was designed as an exploratory feasibility study with a flat outcome structure-having multiple equally-valued outcome measures. Patients were assessed three times individually from week four to week six postoperatively. From week seven to week 12 postoperatively, patients were assessed 12 times during bi-weekly group-based training sessions. They were further assessed 16 and 26 weeks postoperatively (See Fig. 1).

The reporting follows the CONSORT extension for randomized pilot and feasibility trials guidelines [7, 17], as well as the Consensus on Exercise Reporting Template (CERT) [72] (Supporting information (S) 1, S2).

Forty-two patients (cartilage (*n* = 21) or meniscus (*n* = 21) repair) were recruited postoperatively from a referral list at the SOS-R between December 13, 2017 and November 20, 2018. All patients were operated at two hospitals in the Copenhagen Area (Amager-Hvidovre and Bispebjerg-Frederiksberg). The inclusion criteria were patients between 18 and 70 years of age with a cartilage or meniscus repair in one or both knee(s); and who were denied full weight bearing in the entire range of motion of the operated knee joint (See S3). The exclusion criteria were patients being unable to speak or understand Danish or English; orthopedic disorder(s) that required a special rehabilitation program; having a neurological, vascular or cardiac condition; being pregnant; having cancer (current diagnosis); having an active infection; or having a history of a) diagnosed major psychiatric disorder, b) illicit drug use, c) alcohol or medication abuse, d) endothelial dysfunction, peripheral vascular disease, hypertension, diabetes, or e) heart disease and deep vein thrombosis.

Most exclusion criteria (e.g., neurological disease, endothelial dysfunction, peripheral vascular disease, hypertension, diabetes, heart disease and deep vein thrombosis) correspond to the contraindications to BFR-LLST and are derived from a combination of different risk assessment tools to avoid patients experiencing serious events [29, 35, 62].

### Procedures

All patients performed four different types of exercise during their scheduled 9 weeks of rehabilitation at SOS-R (Fig. 1): 1) BFR-LLST supervised, which consisted of three individual and 12 group-based supervised sessions, 2) BFR-LLST home consisted of 30 home-based sessions, 3) usual care (non-BFR) supervised exercise with three individual and 12 group-based supervised sessions; and 4) usual care (non-BFR) exercise at home consisting of 24 home-based sessions. After the scheduled 9 weeks of rehabilitation, the physical therapists responsible for supervised usual care exercise decided whether the patients should continue their rehabilitation.

At each supervised session of BFR-LLST added to usual care exercise (15 sessions in total from week four to week 12 postoperatively), the following acute outcome measures were assessed: adherence, adverse events related to the BFR-LLST, external load during BFR-LLST, knee joint and quadriceps muscle pain before (at rest), during (four sets each) and after (at rest) the BFR-LLST, perceived exertion during BFR-LLST (four sets each), and thigh muscle size indicated by circumference for the operated and healthy leg. Additionally, the maximal knee joint and quadriceps muscle pain during the supervised usual care exercise session was assessed by patients immediately after the session. Additional clinical outcome measures, such as active and passive knee joint range of motion, knee joint effusion, self-reported function and knee-related quality of life, and self-reported functional ability to complete specific activities were recorded at baseline (first individually assessed), and at 16 and 26 weeks postoperatively. At 26 weeks postoperatively, isometric knee extension and flexion muscle strength were assessed. Outcome assessors were experienced physical therapists who were not blinded to the treatment provided. Most of the additional assessments were conducted by the principal investigator (TLJ).

As this study was pragmatic, we were unable to fully control whether the patients received exactly 9 weeks of BFR-LLST added to usual care exercise as listed in the trial registry. An average of 11 weeks is probably more likely because 1) patients were referred and allowed to perform BFR-LLST earlier than expected, and 2) there was often a time delay from last individual BFR-LLST supervised (6 weeks postoperatively) to the first group-based BFR-LLST supervised session (approximately 7 weeks postoperatively) session.

Some between-hospital variation in the content of the rehabilitation regimes existed, but generally the added BFR-LLST knee-extension exercise overheld the following restrictions for patients with cartilage or meniscus repair. BFR-LLST knee-extensions from 90° of knee flexion to full knee extension with no external load were allowed one and 2 weeks postoperatively. At 6 weeks, BFR-LLST knee-extensions from 90° of knee flexion to full knee extension were allowed with external load. Full weight bearing was tolerated from 0 to 90 degrees knee joint flexion from week seven postoperatively (Detailed Rehabilitation regimes, see S3).

### Exercise intervention

#### Blood flow restriction – low-load strength training (BFR-LLST)

The BFR-LLST protocol was the same for patients with cartilage or meniscus repair and is outlined in Table 1 [38, 69, 80]. At baseline, the physical therapist determined the individual patient’s limb occlusion pressure (LOP), which is defined as the minimum occlusion pressure required to stop the flow of arterial blood into the lower limb distal to a pneumatic cuff [58]. This procedure was used to calculate the relative LOP (percentage of LOP) to enhance the treatment effect, while limiting perceived discomfort and risk of adverse events during BFR-LLST [53]. The LOP in the lower limb was identified by increasing the pressure in a 20-cm wide pneumatic cuff with a sphygmomanometer (Heine Gamma® G5, HEINE, Optotechnik GmbH & Co., Herrsching, Germany) around the most proximal part of the thigh until the pulse stopped distal to the cuff, while the patients were sitting on an examination couch with their heel resting on a chair or the floor and their knee joint flexed between 45 and 90 degrees. Distal pulse stop was registered on a finger clip connected to a portable hand-held oximeter attached to the patients’ second toe. This device has proven to be valid and reliable in determining LOP in the lower extremity when compared with a high-resolution Doppler ultrasound scanner [9]. To account for variability associated with the determination of the LOP, the individual patient’s LOP was reduced by 20 mmHg. From this adjusted LOP, 80% LOP that was used in the intervention was calculated. When exercising at very low intensity (20% of 1RM), 80% LOP has been proposed to increase hypertrophy [45] and muscle activity [21] in the quadriceps muscle. After individualizing the pressure, the patients were meticulously instructed in how to perform unilateral BFR-LLST for the knee-extension exercise with a cuff (same as used for LOP determination) at the SOS-R, and at home with an elastic band (Trithon Knee Wraps, Trithon Sport, Denmark) (S2, S4). Patients performed BFR-LLST without external loads for the first 6 weeks postoperatively. Subsequently, BFR-LLST was performed using weight bands fixed around the ankle. At each BFR-LLST supervised session, the external load was increased by between 0.5 and 1.0 kg, if the patient could perform more than 15 repetitions in the fourth and final set. The patients were instructed to perform BFR-LLST at home in the same manner, with the elastic band tightened around the proximal thigh and lifting similar external loads, using weight bands provided by the SOS-R.

**Table 1:** The BFR-LLST protocol for the knee-extension exercise [11, 28, 29]

Variables	Knee-extension exercise
Load, repetition maximum	30 (~ 20–40% of 1RM)
Repetitions per set	≤ 6 weeks: 30, 15, 15, max. 30^a^ > 6 weeks: 30, 15, 15, 15
Sets per session	4
Rest between sets, seconds	30–45
Sessions per week	5
Duration of the experimental period, weeks	Approximately 9 weeks; 3 weeks without and 6 weeks with external loads
Contraction modes, seconds	1 isometric, 2 eccentric, 2 concentric
Rest between repetitions, seconds	0
Time under tension, seconds	≤ 6 weeks: 450 (5 seconds x (30 + 15 + 15 + 30 reps)) > 6 weeks: 375 (5 seconds x (30 + 15 + 15 + 15 reps))
Contraction failure in each set	No, only the last set out of 4^a^
Range of motion, degrees	Max. 90
Rest between training sessions, hours	24 to 48 hours
Anatomical definition of the exercise (exercise form)^b^	Yes
Mode per week	≤ 6 weeks: 3 times supervised in total, 4–5 times at home. > 6 weeks: 2 times supervised, 3 times at home.
Cuff application	As proximal as possible on the lower limb
Cuff type (materials)	20-cm width (Supervised with cuff; Home with elastic band).
Occlusion pressure	Cuff: 80% limb occlusion pressure (LOP). Elastic band: Should be as tight as the cuff corresponding to 80% LOP.
BFR-LLST total training time (total time under tension), seconds	≤ 6 weeks: 585 (450) > 6 weeks: 510 (375)
Duration of the entire BFR-LLST session	Max. 10 minutes twice per week (supervised) and max. 10 minutes 3 times per week at home.

^a^Patients started the BFR-LLST without external load the first six weeks postoperatively due to weight-bearing restrictions and the number of repetitions were limited to 30 in the fourth and final set

^b^An anatomically perfect technique to allow efficient “delivery” of load to the muscle

#### Usual care exercise

At the first treatment (baseline) approximately 3 weeks postoperatively, patients were instructed in a usual care exercise program, which consisted of four strengthening and two range of motion exercises according to the rehabilitation regimes for cartilage or meniscus repair (S2, S5). Patients were instructed to perform these exercises daily at home. Additionally, at baseline, the physical therapist instructed patients in correct gait patterns and how they should manage and control their knee joint pain and swelling. At 6 weeks postoperatively, the usual care exercise was replaced with a new criteria-based usual care exercise program that patients performed at home and at SOS-R (S2, S6). The 60-min new usual care exercise program consisted of a pre-warm-up (10 min), warming-up exercises (10 min), progressive strength training exercises targeting ankle plantar flexors, quadriceps, hamstring, and hip abductor and adductor muscles and balance and flexibility exercises (30–35 min). The unilateral knee-extension progressive strength training exercise to increase quadriceps strength was replaced by the BFR-LLST knee-extension exercise to limit mechanical strain and protect the operated knee joint [70]. We recommended patients to perform BFR-LLST knee-extension (duration 10–15 min) after the usual care exercise program. The traditional progressive strength training exercises were performed to volitional muscular failure with the following descriptors: three sets of 12 repetitions using an intensity of 12 RM and time under tension of 4 s (2 s concentric and 2 s eccentric contraction) [80]. When the patients mastered the strength training exercise in a specific muscle group correctly with acceptable patient-perceived knee symptoms/pain, the exercise was replaced with a more demanding weight-bearing strength training exercise within the same muscle group in the usual care exercise program. The patients were given the program in a printed and digital version (Exorlive, Oslo, Norway) sent via email (S6). We were unable to control the rehabilitation that patients received at the hospital prior to study inclusion. Generally, this consisted of daily basic unloaded strengthening and range of motion exercises comparable with the program received at the first treatment (baseline) in this study.

### Clinical application (adherence)

At each BFR-LLST added to the usual care exercise session, the number of training sessions performed for BFR-LLST (supervised and home), usual care exercise (supervised and at home) and the BFR-LLST descriptors (LOP applied, number of sets, repetitions and external load lifted) were recorded. BFR-LLST descriptors at home were patient-reported via a training diary (S4). Furthermore, the specific exercises performed at each usual care exercise supervised session were noted.

### Harms

At each visit, any adverse events potentially related to BFR-LLST at SOS-R or at home were reported by the patients to the physical therapist responsible for each BFR-LLST supervised session. Patients were interviewed based on a pre-defined and standardized questionnaire of potential adverse events related to BFR-LLST (dizziness, quadriceps muscle pain at rest, knee joint pain at rest, bruising, numbness in the lower leg, subcutaneous bleeding (bruising), cardiovascular or respiratory complaints, deep venous thrombosis or other events) [54]. If a patient experienced clinical signs and symptoms of deep venous thrombosis at home [50], they were urged to contact a medical doctor or the physical therapist at SOS-R immediately (S4). An adverse event was categorized as serious if it caused death, was life-threatening, resulted in persistent or significant disability/incapacity or required inpatient hospitalization according to the European Medicines Agency [18]. A serious adverse event would necessitate a permanent discontinuation of the BFR-LLST intervention. Additionally, all adverse events or complications were documented regardless of their perceived relation to the exercise intervention, operation or occurrences not related to the study. The number of possible adverse events were totaled.

### Outcome measures

Acute outcome measures were recorded at each BFR-LLST supervised session. Thigh muscle size was measured 15 cm proximal to the base of patella using a standard tape to register the circumference of the thigh [19, 32, 33, 79]. The value was recorded to the nearest 0.1 cm. Moreover, knee joint and quadriceps muscle pain was assessed using a 0–100-mm visual analogue scale (VAS-mm) with end points of “no pain” and “worst pain imaginable” [8]. Maximal rating of perceived exertion was measured by the patients’ recall immediately after each BFR-LLST set using the Borg scale ranging from 6 (no exertion at all) to 20 (maximal exertion) [6]. At the baseline, 16 and 26-week assessments, additional outcome measures were recorded. These were: knee joint range of motion measured with a large (30 cm long-armed) universal goniometer [32], and knee joint effusion using a standard tape measure positioned 1 cm proximal to the base of patella [32]. Knee self-reported function and quality of life [67] was assessed using the Knee Injury and Osteoarthritis Outcome Score (KOOS; including subscales of symptoms, pain, activities of daily living, function in sport/recreation, and knee-related quality of life) with scores ranging from 0 to 100 [67]. To address self-reported functional status, we used the patient-specific functional scale (PSFS), where the patients scored on a scale ranging from 0 to 10 their ability to perform self-selected important activities they were unable to do or had difficulty with [77]. The average score of a maximum of five activities was calculated. Both of the self-reported questionnaires were scored on a worst to best scale. At 26 weeks postoperatively, maximal isometric knee extension and flexion strength in 60 degrees of knee flexion were measured using a handheld dynamometer (MicroFet 2, Hoogan Scientific, Salt Lake City, US) placed between the patients’ distal tibia and the resistance pad of a leg extension/leg curl strength training machine. The resistance pad was held in a fixed position by the load of the weight stacks of the training machine [23, 51]. Patients had one practice trial followed by minimum three and maximum 10 knee-extensions with verbal encouragement. The test was ended if the isometric knee extension strength decreased in two consecutive trials or the maximum of 10 trials was reached [1]. The highest strength value was used as the data point. The same procedure was used to determine maximal isometric knee flexion strength.

### Sample size

**Fig. 2:**
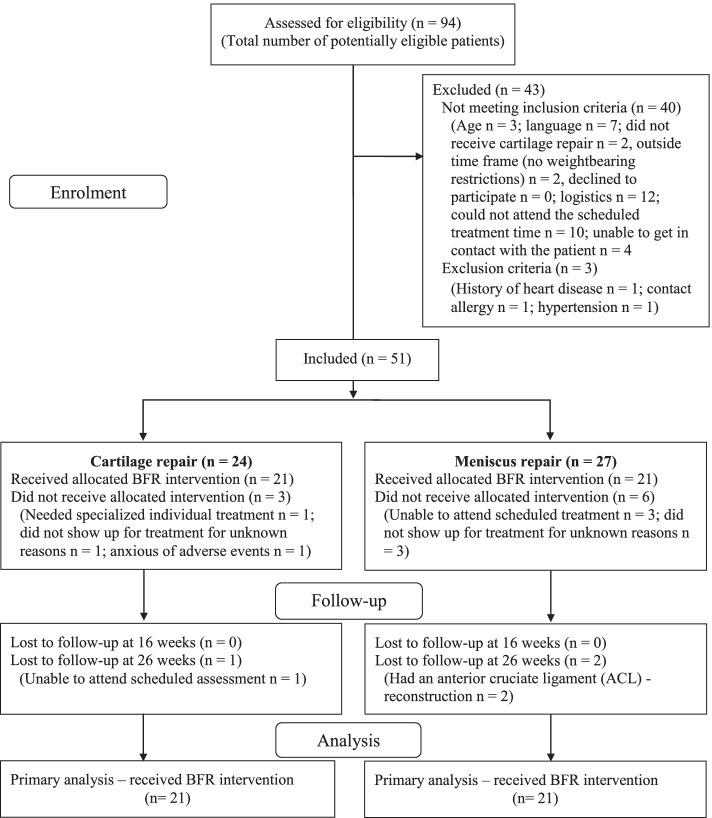
Patient flow diagram

No formal sample size calculation was performed due to the descriptive character of the study and no efficacy testing was performed [2]. Justifications for the sample size in feasibility studies vary greatly [4]. It has been recommended to target a sample size between 15 [34] and 50 [71] for pilot/feasibility studies. We targeted a sample size of 40 patients with a full outcome dataset and continued recruitment until this was achieved (Fig. 2). We aimed for an equal distribution of patients with cartilage (*n* = 20) or meniscus (*n* = 20) repair. We considered a sample of 40 patients large enough to determine the acceptability of the intervention, evaluate the study protocol (for a potential future large-scale trial) and provide enough data for future population-specific sample size estimations [36]. To allow for dropouts and attrition after the final patient in each group was included, we enrolled 51 patients in our study.

### Statistical analysis

We used a linear mixed-effect model to analyze 1) within-group changes for all outcome measures over time from baseline to the different time points (assessments during each BFR-LLST added to the usual care exercise supervised session, 16 and 26-week postoperatively) and 2) within-set changes from the first to fourth set (four sets each) recorded during each BFR-LLST supervised session (15 sessions per patient) in all patients. A type 3 test for the linear mixed-effect model was used to evaluate the overall effect from the first to fourth set during the BFR-LLST supervised session. Changes in knee joint and quadriceps pain at rest from before to after each BFR-LLST supervised session, and the difference in maximal knee joint pain during usual care exercise and the BFR-LLST supervised sessions were examined using a Wilcoxon signed-rank test. No multiple comparisons were performed for any individual timepoints. Assumption of normal distribution was evaluated using Q-Q plots, histograms and Shapiro-Wilk tests. The goodness of model fit to the data was evaluated for normality of residuals and variance homogeneity by residual vs. predicted outcome plots. The percentage of completed training sessions performed was calculated by dividing the performed number of training sessions by the total number of training sessions scheduled expressed as a percent. Missing values in the linear mixed effect models were ignored under the assumption of data being missing at random (MAR) and the likelihood estimation used in the models [43].

All data were entered in EpiData Entry, version 3.3 (Epidata, Odense, Denmark). Analyses were performed using R version 4.1.2 (R Foundation for Statistical Computing, Vienna, Austria), Microsoft Excel programs (Microsoft Office 365, 2019, Redmond, WA, US) and Graphpad Prism version 8.3.1 (GraphPad Software, San Diego, California, US). A *P*-value less than 0.05 was considered statistically significant.

## Results

Ninety-four patients with cartilage or meniscus repair were assessed for eligibility from December 2017 to March 2019 (Fig. 2), which was the total number of potentially eligible patients during that time period. Of those 94 patients, 43 patients were excluded leaving 51 patients eligible for inclusion. Four patients withdrew from the study for reasons unrelated to the BFR-LLST. Four patients did not show up for their treatment, and we were unable to get in contact with them by phone or email. Nothing in their data (complaints, adverse events, complications) indicated that the reason for dropping out was related to the BFR-LLST. One patient dropped out before the BFR-LLST started due to anxiety concerning adverse events. In total, 42 patients with cartilage (*n* = 21) or meniscus (*n* = 21) repair received the BFR-LLST intervention, and characteristics of the patients at baseline, 16 and 26-week assessments are presented in Table 2 and S7 Table 1.

**Table 2:** Baseline characteristics for the patients with cartilage or meniscus repair

Characteristics	Cartilage (***n*** = 21)	Meniscus (***n*** = 21)
Age, mean ± 1SD, years	32 ± 8	28 ± 6
Women/men, n (%)	5 (24)/16 (76)	8 (38)/13 (62)
Body mass index, mean ± 1SD, kg/m^2^	26.1 ± 3.6	24.9 ± 3.5
Smoker, n (%)	4 (19)	5 (24)
Repair type, (n)	Patella (5)/FT (8)/MFC (6)/LFC (2)	LM (5)/MM (15)/LM + MM (1)
Cartilage size damage^a^, median (IQR), cm^2^	2.30 (1.45 to 3.25)	
Multiple-operations^b^, n (%)	12 (57)	11 (52)
Right/left operated knee, n (%)	9 (43)/12 (57)	15 (71)/6 (29)
Same knee operated before, n (%)	10 (48)	7 (33)
Opposite knee operated before, n (%)	6 (29)	4 (19)
Postoperative complications^c^, n (%)	2 (10)	3 (14)
Supervised rehabilitation prior to baseline assessment, n (%)	5 (24)	3 (14)
Tried BFR before baseline assessment, n (%)	5 (24)	5 (24)
Accustomed to exercise, n (%)	18 (86)	18 (86)
Have used pain medication before baseline assessment, n (%)	5 (24)	2 (10)
Acetaminophen (Paracetamol), n (%)	5 (24)	2 (10)
NSAID, n (%)	4 (19)	2 (10)
Opioids and opioid-like drugs, n (%)	2 (10)	1 (5)
Hospital performing the operation, AHH/BBH, n (%)	8 (38)/13 (62)	5 (24)/16 (76)
Time from operation to baseline assessment, median (IQR), weeks	2.0 (1.9 to 5.0)	2.3 (2.0 to 2.6)

*SD* Standard Deviation, *IQR* Interquartile Range, *LM* Lateral meniscus, *MM* Medial meniscus, *FT* Femur trochlea, *MFC* Medial femur condyle, *LFC* Lateral femur condyle, *BFR* Blood flow restriction, *NSAID* Non Steroidal Anti-Inflammatory Drug, *AHH* Amager-Hvidovre Hospital, *BBH* Bispebjerg Hospital

^a^*n* = 20

^b^Anterior cruciate ligament (ACL)-reconstruction (*n* = 6)

^c^Knee stiffness, muscle tightness, knee pain, quadriceps hematoma

### Clinical application (adherence) and training characteristics

Patients with cartilage or meniscus repair completed on average more than 80% of the BFR-LLST supervised, BFR-LLST at home, usual care exercise supervised and usual care exercise at home (S7 Table 2). On average, the BFR-LLST added to the usual care exercise intervention period lasted 11 (SD ± 1.2) weeks. The individualized limb occlusion pressure (LOP) applied varied from 104 to 176 mmHg during the BFR-LLST supervised, and on average the patients performed the recommended 75 repetitions per BFR-LLST supervised session approximately 79% of the time (S7 Table 2). The median external load during the BFR-LLST knee-extension exercise increased from 0 kg at baseline to 2.5 kg at the end of the BFR-LLST supervised intervention period (median = 0.28 kg/week, range = 0.23–0.32 kg/week, *p* < 0.001) (S7 Table 2, S7 Table 3), but overall remained low during the intervention (median = 1 kg, range = 0–7 kg) (S7 Table 2). The number and name of the exercises performed during the group-based usual care exercise supervised program at SOS-R are shown in S7 Table 4.

### Outcome measures

#### Knee joint and quadriceps muscle pain

Using the classification by the International Association for the Study of Pain (IASP), patients experienced none to mild [81] maximal knee joint pain during BFR-LLST supervised (median VAS-mm = 0, IQR = 0–12), which was significantly lower to a clinically meaningful [16, 20] degree than the maximal knee joint pain experienced during usual care exercise supervised (median VAS-mm =23, IQR 0–40) (*p* < 0.001) (S7 Table 2). On the contrary, patients experienced moderate to severe maximal quadriceps muscle pain during BFR-LLST supervised (median VAS-mm = 47, IQR 12–70), which was significantly higher to a clinically meaningful degree [16] than the maximal quadriceps muscle pain during usual care exercise supervised (median VAS-mm = 0, IQR 0 to 6) (*p* < 0.001). The median value of the knee joint and quadriceps muscle pain at rest before and after BFR-LLST supervised session were zero (all median VAS-mm = 0) (S7 Table 2).

#### Changes during the BFR-LLST intervention period

**Fig. 3:**
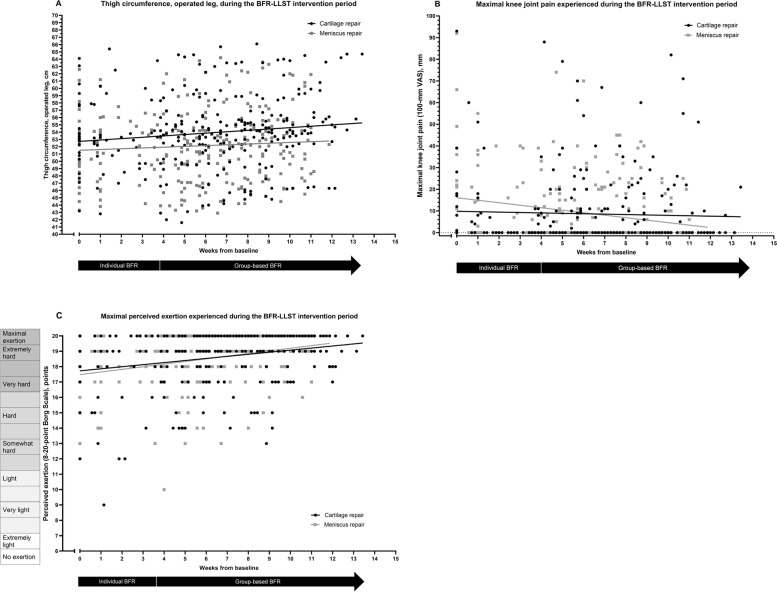
Scores of each patient and mean scores for the patients with cartilage (black dots and line) or meniscus repair (gray dots and line) over time for thigh circumference (**A**), knee joint pain (**B**) and perceived exertion (**C**) during the BFR-LLST intervention. BFR-LLST; Blood flow restriction – low load strength training. VAS; Visual Analogue Scale

During the 11-week BFR-LLST intervention period, the thigh circumference of the operated leg, increased an average of 0.12 cm per week in the meniscus repair group (*p* < 0.001), and a similar pattern (although not statistically significant) was found in the cartilage repair group (0.05 cm per week, *p* = 0.099) (Fig. 3A, S7 Table 3). In comparison with the thigh circumference of the healthy leg, the difference between legs diminished over time in the cartilage (− 0.06 cm per week, *p* < 0.006) and meniscus groups (− 0.10 cm per week, *p* < 0.001) (S7 Table 3). Maximal quadriceps muscle pain did not change over time for any patients (*p* = 0.330), while maximal knee joint pain decreased over time in the meniscus repair group only (− 1.1 VAS-mm per week, *p* < 0.010) (Fig. 3B, S7 Table 3). Patients found the BFR-LLST to be slightly more demanding at the end compared to the beginning of the intervention period, as the rating of perceived exertion (Borg Scale) increased with approximately 0.1 and 0.2 points per week for the cartilage repair group (*p* < 0.034) and meniscus repair group, respectively (*p* < 0.001) (Fig. 3C, S7 Table 3).

#### Changes within the BFR-LLST supervised session (four sets each)

**Fig. 4:**
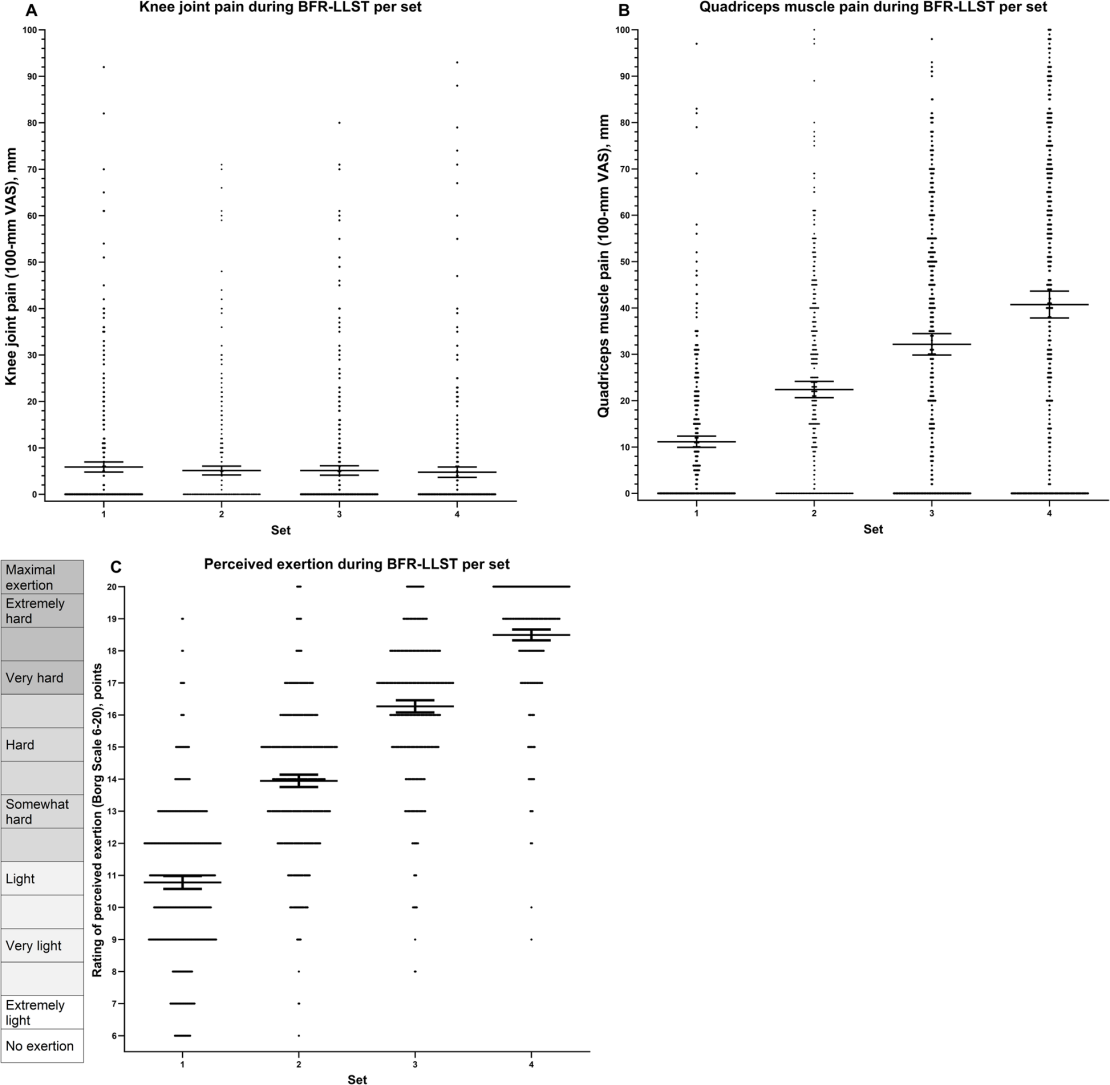
Knee joint pain (**A**), quadriceps muscle pain (**B**) and perceived exertion (**C**) from the first to the fourth set within the BFR-LLST supervised session (15 sessions each) for all patients. Scatterplots with means including whiskers representing 95% confidence intervals

On average, patients experienced none to mild (4.8 to 5.9 VAS-mm) knee joint pain and no difference between the first to the fourth set of BFR-LLST (*p* = 0.224) (Fig. 4A, S7 Table 6). On the contrary, increases were found from the first to the fourth set for quadriceps muscle pain (Fig. 4B, S7 Table 6) and rating of perceived exertion (Borg Scale) (Fig. 4C, S7 Table 6) from 11.1 to 40.7 VAS-mm (*p* < 0.0001) and from10.8 to 18.5 points (*p* < 0.0001), respectively.

#### Clinical outcome measures

Changes in clinical outcome measures from baseline to 16 and 26 weeks postoperatively in patients with cartilage or meniscus repair are presented in Table 3. Significant improvements were seen from baseline to 16 or 26 weeks postoperatively for thigh circumference (operated leg), KOOS subscales, PSFS and active/passive knee joint range of motion of extension/flexion. No significant increase in knee joint effusion in the operated leg was seen from baseline to 26 weeks. At 26 weeks postoperatively, an isometric knee-extension muscle strength deficit of the operated compared to the healthy leg (Limb symmetry index (LSI) = 76–89%) existed. This isometric muscle strength deficit was small for knee-flexion (LSI = 94–96%).

**Table 3:** Outcome measures at baseline, 16 and 26-week assessment

Group	Baseline		16 weeks		Difference (16 weeks – Baseline)		26 weeks		Difference (26 weeks – Baseline)	
	Cartilage (*n* = 21)	Meniscus (*n* = 21)	Cartilage (*n* = 21)	Meniscus (*n* = 21)	Cartilage^a^ (*n* = 21)	Meniscus (*n* = 21)	Cartilage (*n* = 20)	Meniscus (*n* = 19)	Cartilage (*n* = 20)	Meniscus (*n* = 19)
Thigh circumference, operated, cm	53.7 ± 5.3	51.8 ± 5.2	54.5 ± 5.2	53.4 ± 5.0	0.8 ± 1.3**	1.6 ± 1.2***	55.2 ± 5.0	53.5 ± 4.7	1.0 ± 1.3***	1.8 ± 2.3***
Thigh circumference, healthy, cm	56.0 ± 4.9	53.8 ± 4.4	56.5 ± 4.9	54.7 ± 4.6	0.5 ± 1.2*	0.9 ± 0.8**	56.9 ± 4.9	54.5 ± 4.3	0.4 ± 1.3	0.9 ± 1.5**
Thigh circumference difference (healthy – operated leg), cm	2.3 ± 1.9	2.0 ± 1.4	2.0 ± 1.5	1.3 ± 1.3	-0.3 ± 1.5*	-0.7 ± 1.2*	1.7 ± 1.4	1.0 ± 1.4	-0.7 ± 1.4***	-0.9 ± 1.5**
KOOS subscale scores, points										
Pain	54.2 ± 21.0	51.7 ± 16.9	78.2 ± 14.0	81.1 ± 13.9	23.2 ± 15.6***	29.4 ± 20.2***	79.3 ± 16.4	78.8 ± 19.2	24.8 ± 18.9***	27.7 ± 23.3***
Symptoms	53.7 ± 16.8	52.7 ± 14.0	76.3 ± 11.0	74.1 ± 16.5	22.1 ± 21.7***	21.4 ± 13.9***	79.5 ± 12.3	75.0 ± 22.7	25.4 ± 23.7***	22.4 ± 16.5***
Activities of daily living	49.4 ± 26.8	54.2 ± 20.6	83.4 ± 13.2	86.5 ± 12.1	32.5 ± 20.2***	32.3 ± 21.9***	87.7 ± 13.7	86.5 ± 16.8	37.4 ± 23.3***	33.0 ± 22.1***
Sports/recreation	4.5 ± 6.7	6.7 ± 9.7	25.3 ± 17.1	40.2 ± 23.0	20.5 ± 17.2***	33.6 ± 21.2***	42.3 ± 23.6	55.8 ± 31.5	37.5 ± 23.5***	49.5 ± 29.9***
Quality of life	26.5 ± 17.1	34.5 ± 18.4	34.7 ± 18.3	43.8 ± 22.6	7.5 ± 13.5**	9.2 ± 17.9*	45.6 ± 19.0	49.3 ± 22.1	18.4 ± 16.9***	14.1 ± 21.0**
Patient-Specific Functional Scale (PSFS), points	0.9 ± 0.9	1.1 ± 1.2	4.6 ± 2.1	5.4 ± 2.0	3.7 ± 2.2***	4.3 ± 2.3***	5.49 ± 2.0	6.7 ± 2.0	4.6 ± 2.0***	5.7 ± 2.2***
Knee joint effusion, operated, cm	43.1 ± 4.3	41.5 ± 3.4	42.5 ± 4.2	41.3 ± 3.3	-0.6 ± 0.8*	-0.2 ± 1.2	43.1 ± 3.4	41.0 ± 3.0	-0.5 ± 1.3*	-0.2 ± 1.6
Knee joint effusion, healthy, cm	42.6 ± 4.0	40.9 ± 3.1	43.0 ± 4.2	41.6 ± 2.9	0.4 ± 0.6*	0.6 ± 0.7***	43.3 ± 3.5	41.5 ± 2.9	0.2 ± 0.8	0.7 ± 0.9***
Knee AROM flexion, degrees	122.0 ± 13.7	92.8 ± 28.7	138.1 ± 6.7	140.1 ± 8.7	16.1 ± 14.1***	47.4 ± 30.9***	138.6 ± 7.7	140.4 ± 9.1	16.7 ± 14.3***	48.1 ± 29.4***
Knee PROM flexion, degrees	128.2 ± 15.8	95.3 ± 28.0	144.4 ± 6.8	145.1 ± 8.3	16.2 ± 15.3***	49.8 ± 29.4***	144.4 ± 6.3	145.9 ± 8.7	16.4 ± 15.4***	51.8 ± 29.0***
Knee AROM extension, degrees	-4.2 ± 5.8	-2.5 ± 6.5	-7.6 ± 4.5	-6.9 ± 6.2	-3.3 ± 4.2***	-4.4 ± 6.5***	-7.1 ± 4.3	-9.0 ± 5.1	-3.0 ± 4.5**	-7.0 ± 5.4**
Knee PROM extension, degrees	-9.1 ± 5.2	-5.2 ± 6.4	-10.6 ± 4.9	-10.0 ± 7.1	-1.5 ± 2.8*	-4.8 ± 7.5**	-10.1 ± 3.9	-11.9 ± 5.3	-1.1 ± 3.0	-7.2 ± 6.1*
Knee-extension strength, operated, Nm/kg							1.78 ± 0.63	1.99 ± 0.67		
Knee-extension strength, healthy, Nm/kg							2.33 ± 0.52	2.29 ± 0.59		
Limb strength index, knee-extension strength, %							76 ± 20	89 ± 29		
Knee-flexion strength, operated, Nm/kg							1.26 ± 0.33	1.37 ± 0.33		
Knee-flexion strength, healthy, Nm/kg							1.37 ± 0.36	1.46 ± 0.40		
Limb strength index, knee-flexion strength, %							94 ± 18	96 ± 19		

All values are presented as mean ± SD

Negative knee extension scores indicate ROM scores, where the knee extends more than the 0° starting position. Nm/kg = Newton × meter/kg. Limb strength index =  Strength, operated/strength, healthy) × 100%

Abbreviations: *KOOS* Knee Injury and Osteoarthritis Outcome Score, *AROM* active range of motion, *PROM* passive ROM

^*^p < 0.05

^**^p < 0.01

^***^p < 0.001

^a^KOOS subscale scores *n* = 20

### Harms

Thirty-eight of the 41 patients reported a total of 146 adverse events, but none were considered serious (S7 Table 5). Dizziness (52 cases) was experienced by approximately half of the patients (*n* = 19) - but no one fainted. All the adverse events were considered transient, meaning the symptoms disappeared within a short time frame after the completion of the BFR-LLST session, and no patients dropped out because of BFR-LLST.

At the 26 weeks postoperative assessment, five out of 21 (25%) and eight of 19 (42%) of patients with cartilage or meniscus repair, respectively, reported postoperative complications (S7 Table 1). Knee joint pain was the most prevalent reported complication, which seemed to be related to the initiation of weight-bearing or impact activities.

## Discussion

The main findings were that 9 weeks of BFR-LLST added to usual care exercise initiated early after cartilage or meniscus repair in the knee joint seemed feasible as patients adhered well to the BFR-LLST protocol without any related reports of serious adverse events or exacerbation of knee-related symptoms (e.g. pain). No disuse atrophy of the thigh muscles (muscle mass) was found, despite patients having restrictions on weight-bearing in the intervention period.

## Interpretation

### Clinical application (adherence) and training characteristics

Patients were able to adhere to the high-volume (five times a week) high-intensity (80% LOP) BFR-LLST protocol, using both a pneumatic cuff during BFR-LLST supervised and a simple elastic band at home (BFR-LLST home). It should be noted that two patients had the relative LOP reduced either transiently or permanently due to discomfort. As a result, adjustment of the relative LOP must be considered in patients experiencing discomfort from a tight pneumatic cuff around the proximal thigh, even though little difference in discomfort during BFRT elbow flexion with relative LOPs ranging from 40% to 90% has been reported in healthy individuals [46]. Only one of nine dropouts in the study was related to BFR-LLST. The patient was anxious about potential harms of BFR-LLST and dropped out prior to the first BFR-LLST supervised session at baseline.

### Harms

According to the current literature, reporting of adverse events in a standardized and systematic way in randomized controlled trials examining exercise therapy [57] or BFR exercise [27] is rare. The focus is typically efficacy of new interventions, such as BFR exercise [31]. A systematic approach to registering adverse events seems imperative as serious and non-serious adverse events and concerns related to BFR exercise have been reported in surveys of practitioners or individuals [54, 61]. Therefore, we conducted a prospectively registered study examining patients with weight-bearing restrictions that focused on, and registered, adverse events related to BFR-LLST at each supervised session. Like other studies [29, 79], we did not find any serious adverse events [18], although the study was very likely underpowered to detect rare serious adverse events. However, we noted a higher frequency of transient dizziness compared to other clinical trials [29, 54, 79] but similar to the experience of practitioners of BFR-LLST [61]. Half of the patients experienced dizziness while loosening the cuff after the BFR-LLST, which may be associated with hypotension or a vaso-vagal response [61]. We registered very few events of bruising or subcutaneous hemorrhage compared to a large survey of healthy individuals performing BFR exercise [54]. Importantly, patients experienced more frequent itching of the lower leg, calf tightness and discoloration of the leg distal to the cuff. Even though no serious adverse events were registered, it should be stressed that risks (e.g., neurological complications, thrombotic events) of using a pneumatic tourniquet–assisted system have been found in patients undergoing lower limb surgery [65, 86]. Therefore, practitioners are strongly encouraged to follow risk assessment tools [29, 35, 62], manage careful patient selection, and possess a high level of proficiency in applying BFR-LLST, to preclude patients experiencing any serious adverse events related to BFR-LLST after knee surgery. Finally, at the 26-week assessment, the patient-reported postoperative complications were surprisingly high in patients with meniscus repair, which is frequent and not related to the rehabilitation protocols [55].

### Outcome measures

#### Knee joint pain, quadriceps muscle pain and perceived exertion

Clinicians and orthopedic surgeons have concerns about knee joint pain and intra-articular damage during postoperative rehabilitation [70]. The maximal of knee joint pain experienced during the BFR-LLST was close to none, and lower than during usual care exercise, which supports BFR-LLST for patients with cartilage or meniscus repair that require prolonged weight-bearing restrictions [39, 70]. The reason for the lower pain levels could be the lower external load on the knee joint during BFR-LLST knee-extension and/or the BFR-LLST-induced hypalgesic effect [37]. The maximal quadriceps muscle pain experienced during BFR-LLST was moderate and higher compared to the minimal pain experienced during usual care exercise. This moderate quadriceps muscle pain is also reported in patients performing BFR-LLST early after ACL (anterior cruciate ligament) reconstruction [28]. The perceived pain increase was temporary during the occlusion period, as knee joint and quadriceps muscle pain at rest before and after the BFR-LLST session was close to none, indicative of no exacerbation of pain symptoms over time.

As seen in healthy subjects performing BFR exercise [13, 84], patients experienced the BFR-LLST becoming, on average, gradually more painful (mild to moderate) in the quadriceps muscle and demanding (light to very/extremely hard) from the first to the fourth set. The gradual reduction of blood flow to the quadriceps muscle during BFR-LLST impacts tissue metabolism and fatigue [26, 78], which may explain the perceived quadriceps muscle pain [22] and exertion [26]. Interestingly, some patients in the present study did not register quadriceps muscle pain. This finding may be explained by multiple reasons. Some patients associated quadriceps muscle pain during BFR-LLST (exercise-related) as beneficial to enhance their recovery [14], or/and b) they had a high tolerance for muscle pain during exercise [52].

Patients experienced the same or a slightly higher perceived exertion during the BFR-LLST over the entire intervention period, indicating that the BFR-LLST stimulus was constant as the external training load (kg lifted) during BFR-LLST knee-extension increased. Additionally, quadriceps muscle and knee joint pain remained stable or tended to decrease during the intervention period. Similar patterns were found in patients with ACL-reconstruction over an eight-week BFR intervention period, which suggests that BFR-LLST might have a hypalgesic effect on knee joint pain [28]. The mechanism behind the reduction of knee joint pain during BFR-LLST is unclear, but the moderate quadriceps muscle pain originating from the ischemic and exercise-induced and/or the limb occlusion pressure during BFR-LLST may have had a pain modulating effect in the present study [37].

#### Thigh circumference, muscle strength and additional clinical outcomes

Thigh circumference (and the difference in thigh circumference between legs) as surrogate proxy for thigh muscle mass did not decrease during the intervention period, even though patients followed an early range of motion and weight-bearing restrictions protocol after cartilage or meniscus repair. This is in line with research showing that BFR-LLST helps to prevent muscle disuse atrophy in patients after knee surgery [78, 79] and healthy individuals [10, 44, 73]. To our knowledge, no study has indicated the beneficial effect of BFR-LLST (or any other intervention) on disuse atrophy performed early after cartilage or meniscal repair without exacerbation of knee joint and quadriceps muscle pain. Still, we found a knee-extension muscle strength deficit of the operated compared to the non-operated leg (LSI) 26 weeks postoperatively in patients with cartilage or meniscus repair. Several factors may explain the knee-extension deficit after knee surgery. First, the scheduled BFR-LLST dose with four sets at each BFR-LLST session, five times a week for 9 weeks were maybe not sufficient to counteract the negative disuse effects in the early postoperative phase with limited mobilization and weight-bearing. Second, the traditional knee-extension progressive high-load strength training was not part of the late rehabilitation program (at 6 weeks postoperatively). This exercise may have increased the knee-extension strength more compared to BFR-LLST knee-extension [27], but potentially increases the number of postoperative complications [70, 75]. Third, complications after cartilage [83] or meniscus [5, 42] repair are common and may have reduced the patients’ willingness to perform maximal knee-extension strength tests. Fourth, residual levels of arthrogenic inhibition of the knee extensors may have persisted 26 weeks postoperatively [3].

Overall, our KOOS scores were slightly better (five to 15 points higher on KOOS pain, KOOS symptoms and KOOS ADL), and our knee-extension strength showed similar values (S7 Table 7), to results derived from prospective studies investigating either cartilage repair [68, 82] or meniscus repair with [41] or without [64] ACL reconstruction surgery, between 6 months to 2 years postoperatively. However, KOOS scores, especially the subgroups KOOS Sport/recreation and KOOS Quality of life, remained up to 25–30 points lower 6 months postoperatively than a reference population [60]. Our clinical outcomes (KOOS and lower limb strength) require a long-term rehabilitation strategy after cartilage [82] or meniscus repair [64].

## Study limitations

Several limitations should be noted. First, the study was an unblinded prospective feasibility study without a control group. Therefore, we cannot determine the efficacy of early BFR-LLST after cartilage or meniscus repair. A larger blinded randomized controlled trial should be undertaken to test the hypothesis that BFR-LLST added to usual care exercise is superior to usual care alone, for knee-extension strength and physical function after cartilage or meniscus repair. Second, no objective registration was carried out to measure patients’ adherence to the BFR-LLST protocol at home. However, we stressed the importance of adhering to the BFR-LLST home program and patients were encouraged to fill out their patient-reported training diary at each visit, which we believe resulted in the high reported adherence rate. Third, we had no pre-surgery records, and were unable to control the intervention at the rehabilitation center prior to inclusion in the study. After inclusion, patients followed the standardized rehabilitation program. Fourth, the results of this study cannot be extrapolated to other BFR-LLST protocols. However, our BFR-LLST protocol was similar to the recommended BFR-LLST protocols to enhance muscle mass and strength [49, 62, 69]. Fifth, the relative restrictive stimulus (percentage of LOP) is influenced by diurnal variability in the systolic blood pressure [30]. To ensure a more accurate stimulus, multiple LOP measurements prior to the BFR-LLST over time may have provided a more valid intended relative stimulus (80% LOP).

## Clinical applications (generalizability)

The study was an explorative prospective feasibility study, in which patients followed a usual care exercise pathway in a clinical practice setting, but BFR-LLST knee-extension without load was added to the program from week 4-6 week postoperatively and BFR-LLST knee-extension with external load replaced the traditional progressive strength training from week seven to 12 postoperatively. If a clinician has a patient that fulfils the exclusion criteria and takes heed of the patient’s feedback at each supervised BFR-LLST session, we believe the methods used in this study can be transferred directly into clinical practice, as the study was conducted in a clinical setting, for patients with early weight-bearing restrictions after cartilage or meniscus repair of the knee joint. It should be noted that patients were primarily young and had free access to their treatment.

## Conclusion

Nine weeks of BFR-LLST added to usual care exercise initiated early after cartilage or meniscus repair in the knee joint seemed feasible without exacerbation of knee joint symptoms. Patients adhered well to the BFR-LLST protocol. Adverse events were reported, for example dizziness, in most patients, but none were considered serious. No disuse atrophy of the thigh muscles was found, despite patients having weight-bearing restrictions in the intervention period. The encouraging results call for a RCT to investigate the efficacy of early BFR-LLST in patients with similar or a higher level of disability.

## Supplementary Information

**Additional file 1: S1.** Checklist CONSORT extension to randomized pilot and feasibility trials.

**Additional file 2: S2.** CERT (Consensus on Exercise Reporting Template).

**Additional file 3: S3.** Rehabilitation regimens - Cartilage (Steadman procedure) or meniscus repair.

**Additional file 4: S4.** Blood Flow Restriction Exercise Leaflet.

**Additional file 5: S5.** Usual care exercise after cartilage or meniscus repair in the knee joint - week 3–6 postoperatively.

**Additional file 6: S6.** Usual care exercise after cartilage or meniscus repair in the knee joint - week 7 postoperatively.

**Additional file 7: S7.** Supplementary results. **S7 Table 1.** Patient characteristics at 16 and 26-week assessment. **S7 Table 2.** Clinical application (adherence), training characteristics and pain at rest and during BFR-LLST added to usual care exercise. **S7 Table 3.** Change per week in thigh circumference, knee joint and quadriceps pain, perceived exertion and training load during the, on average, 11 weeks of BFR-LLST added to usual care exercise intervention period (15 sessions). **S7 Table 4.** Number of exercises performed during the group-based usual care exercise supervised program (15 sessions). **S7 Table 5.** Adverse events during the BFR-LLST added to usual care exercise intervention period. **S7 Table 6.** Average and overall change in knee joint pain, quadriceps muscle pain and perceived exertion from 1st to 4th set within the BFR-LLST session (15 sessions) for all patients (*n* = 42).

## Data Availability

The datasets used and/or analyzed during the current study are available from the corresponding author on reasonable request.

## Abbreviations

BFR-LLST: Blood flow restriction – low load strength training
RCT: Randomized controlled trial
RM: Repetition maximum
SOS-R: Section for Orthopedic and Sports Rehabilitation
CERT: Consensus on exercise reporting template
S: Supporting information
LOP: Limb occlusion pressure
VAS: Visual analogue scale
KOOS: Knee injury and osteoarthritis outcome score
PSFS: Patient-specific functional scale
MAR: Missing at random
ACL: Anterior cruciate ligament
SD: Standard deviation
IQR: Interquartile range
LM: Lateral meniscus
MM: Medial meniscus
FT: Femur trochlea
MFC: Medial femur condyle
LFC: Lateral femur condyle
NSAID: Non-steroidal anti-inflammatory drug
IASP: International association for the study of pain
LSI: Limb symmetry index
