# Supplementary material for: Blood flow restriction added to usual care exercise in patients with early weight bearing restrictions after cartilage or meniscus repair in the knee joint: a feasibility study
Source: J Exp Orthop. 2022 Oct 4;9:101. doi: 10.1186/s40634-022-00533-4 (PMC9530077; doi:10.1186/s40634-022-00533-4)
Supplement: Supplementary file 2 — Additional file 2: S2. CERT (Consensus on Exercise Reporting Template). [file 40634_2022_533_MOESM2_ESM.docx]

## 1. Type of exercise equipment

Initially, all patients received a written description of the BFR-LLST protocol explaining the advantages, disadvantages and potential risks related to the BFR-LLST (S4 Blood Flow Restriction Exercise Leaflet) intervention. Furthermore, the patients received hand-outs with two different exercise programs for i) the immobilization period week 3-6 (S5), and ii) group session period 7-12 (S6), respectively. The exercise template used during group-based usual care exercise supervised sessions were also used for the home training in the same period of as well as the following period from week 13. All exercises were illustrated with pictures and a thorough written description (S6).

List of training equipment needed during the BFR-LLST added to the usual care exercise program:

- - 20-cm wide pneumatic cuff with a sphygmomanometer attached, 41-59 cm circumference, Heine Gamma® G5, HEINE, Optotechnik GmbH & Co., Herrsching, Germany.
  - An elastic band (Trithon Knee Wraps, Trithon Sport, Denmark) for BFR-LLST home-based.
  - Weight band(s) fixated around the ankle with loads of 0.5, 1, 1.5, 2, 3, 4, 5, 6, 7 kilograms
  - A spinning bike
  - A Concept 2^®^ rowing machine
  - Carpet tiles
  - Variation of dumbbells or kettlebells x 2 to match the exact strength level of the patient
- Barbell with plates matching the strength level of the patient
- Ball (diameter ≈22 cm)
- Body bar
- Theraband^®^ elastic band sheet in green or red colour
- Reebok^®^ step box
- Seated leg press machine (Cybex® VR3, Medway, MA, USA)
- BOSU^®^ - balance trainer (Diameter 65 cm)
- Wobblesmart^®^ balance board

## 2. Instructor qualifications

All physical therapists had a minimum two years of experience with rehabilitation of patients with cartilage or meniscus repair. The physical therapists were familiar with BFR-LLST for patients with weight-bearing restriction and/or knee joint pain. To ensure standardization of the BFR-LLST added to the usual care exercise program, the primary investigator and the clinical responsible physical therapist held a 2-hour instruction session with all physical therapists that carried out the group-based usual care exercise supervised sessions.

## 3. Individual or group

Patients had three individual visits during the first six weeks postoperatively. From approximately week seven postoperatively, patients attended at bi-weekly group-based BFR-LLST added usual care exercise supervised program (15 sessions).

## 4. Supervision

Handouts of all exercise programs and instructions were handed to the patients at the study site. The patients performed the exercises at the Section for Orthopaedic and Sports Rehabilitation (SOS-R) Copenhagen as well as at home.

Twelve group-based sessions were planned for the six-week period (BFR-LLST added to usual care supervised). The group-based sessions included up to eight patients per class. The sessions lasted 60 minutes each and were held twice per week with one or two days in between. The patients started participating in the group-based BFR-LLST added to usual care exercise supervised session after the end of their immobilization period (six weeks postoperatively). Additionally, the patients were instructed to perform the exercises once per week at home or at a gym on the weekends (never training on two consecutive days).

## 5. Adherence

At each group-based BFR-LLST added to usual care exercise supervised session two days a week (from week 7 – 12), the physical therapist registered, which exercises and how many repetitions, sets and the load the individual patient performed. Furthermore, the patients were asked to registerer number of sets, repetitions, and load of each exercise, when they performed the exercises at home, and bring the data to the following group session, so it could be registered by the physical terapist

Patients registered the date, cuff/elastic band, load, sets and number of repetitions and any complaints during the BFR-LLST at home in a training diary in the BFR-LLST leaflet (S4). A physical therapist registered the same data during the BFR-LLST supervised session at the SOS-R.

## 6. Motivation

Generally, the physical therapists encouraged patients to self-manage their rehabilitation and the importance of adhering to the BFR-LLST added to usual care exercise program, and through group dynamics from week 7 to week 12 postoperatively.

## 7. Progression decision rules

Exercises were progressed if patients were able to perform the number of repetitions maximum (RM) and form/quality described in the usual care exercise after cartilage or meniscus repair in the knee joint - week 7- postoperatively template (S6). If the patients were able to complete all three sets with correct form and quality, the patients were progressed to the next exercise level for the specific muscle group. The patients did not progress, if the number of repetitions were not reached or if form/quality were not obtained as described.

If patients were not able to perform the next exercise as described in the template, because of pain or lack of form, more resistance was added to the previous exercise, to ensure progression. The resistance applied in each exercise was registered and used in the next training session at home.

## 8. Exercise description

All patients had four different types of exercises during their scheduled nine weeks of rehabilitation period at SOS-R (Figure 1); 1) BFR-LLST supervised, which consisted of three individual and 12 group-based supervised sessions; 2) BFR-LLST home consisted of 30 home-based sessions; 3) Usual care exercise supervised was three individual and 12 group-based supervised sessions; and 4) Usual care exercise home consisted of 24 home-based sessions. After the BFR-LLST added to usual care exercise supervised intervention period, the physical therapists responsible for the usual care exercise supervised decided whether the patients should continue their rehabilitation.

The usual care exercise programs can be found in S5 and S6. The BFR-LLST protocol can be found in the table (see Table 1) and instructions are found in S4.

All exercises were illustrated with photographs and a thorough written description including number of sets, repetitions, and time under tension.

Figure 1.


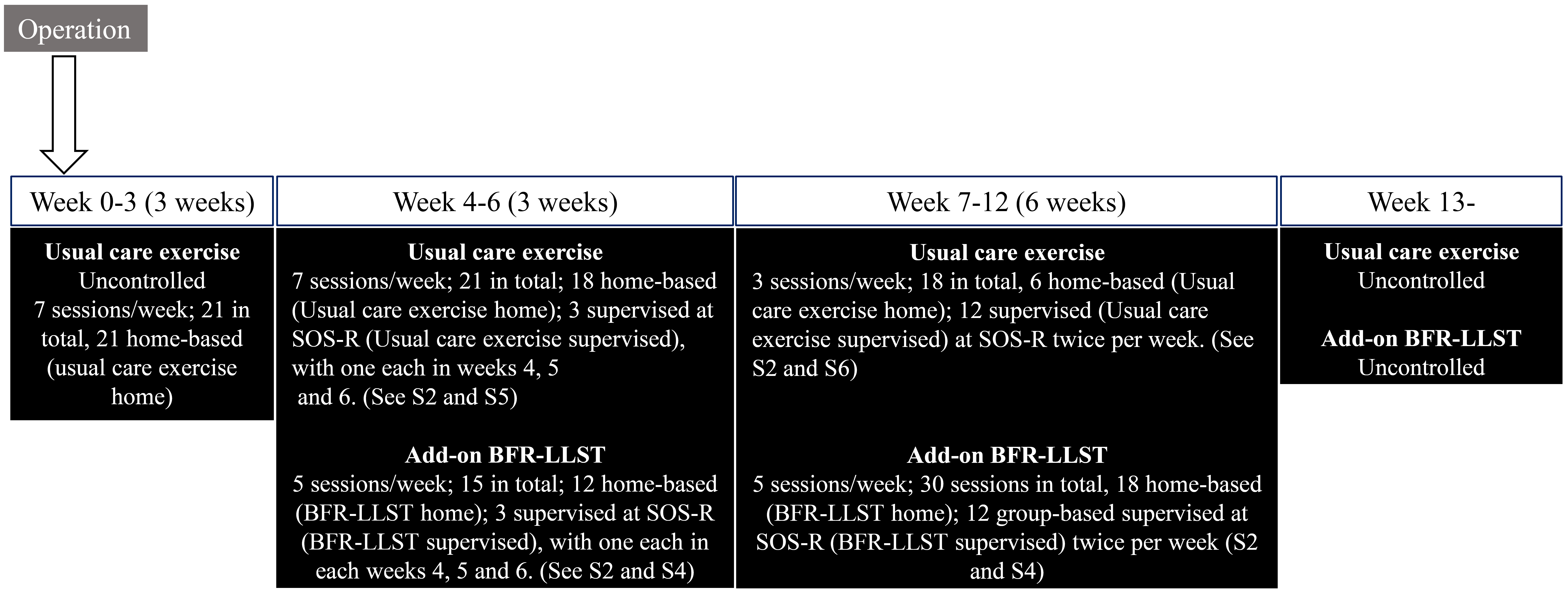


Table 1 The BFR-LLST protocol for the knee-extension exercise

| **Variables** | **Knee-extension exercise** |
| --- | --- |
| Load, repetition maximum | 30 (~20-40 % of 1RM) |
| Repetitions per set | ≤ 6 weeks: 30, 15, 15, max. 30*  > 6 weeks: 30, 15, 15, 15 |
| Sets per session | 4 |
| Rest between sets, seconds | 30-45 |
| Sessions per week | 5 |
| Duration of the experimental period, weeks | Approximately 9 weeks; 3 weeks without and 6 weeks with external loads |
| Contraction modes, seconds | 1 isometric, 2 eccentric, 2 concentric |
| Rest between repetitions, seconds | 0 |
| Time under tension, seconds | ≤ 6 weeks: 450 (5 seconds x (30+15+15+30 reps))  > 6 weeks: 375 (5 seconds x (30+15+15+15 reps)) |
| Contraction failure in each set | No, only the last set out of 4* |
| Range of motion, degrees | Max. 90 |
| Rest between training sessions, hours | 24 to 48 hours |
| Anatomical definition of the exercise (exercise form) | Yes |
| Mode per week | ≤ 6 weeks: 3 times supervised in total. 4-5 times at home.  > 6 weeks: 2 times supervised, 3 times at home. |
| Cuff application | As proximal as possible on the lower limb |
| Cuff type (materials) | 20-cm width (Supervised with cuff; Home with elastic band). |
| Occlusion pressure | Cuff: 80% limb occlusion pressure (LOP). Elastic band: Should be as tight as the cuff corresponding to 80% LOP. |
| BFR-LLST time (total) per session, seconds | ≤ 6 weeks: 585 (9 ¾ min.) (450 time under tension + 3x45 rest)  > 6 weeks: 510 (8 ½ min.) (375 time under tension + 3x45 rest) |
| Duration of the entire BFR-LLST session | Max. 10 minutes twice per week (supervised) and max. 10 minutes 3 times per week at home. |

*Patients started the BFR-LLST without external load the first 6 weeks postoperatively due to weight-bearing restrictions and the number of repetitions were limited to 30 in the 4^th^ and last set.

## 9. Home program

Initially, all patients received a written description of the BFR-LLST leaflet (S4) explaining the advantages, disadvantages and potential risks related to the BFR intervention. Furthermore, the patients received hand-outs with two different home exercise programs for i) individual session week 3-6 postoperatively (S5), and ii) group session period week 7-12 postoperatively (S6), respectively. The exercise templates used during group-based usual care exercise supervised sessions were also used for the home training in the same period of as well as the following period from week 13.

## 10. Non-exercise components

At the first individual BFR-LLST added to usual care exercise session (baseline), the physical therapist instructed patients in correct gait patterns and how they should manage and control their knee joint pain and swelling. Other non-exercise components were non-standardized.

## 11. Adverse events.

The BFR-LLST leaflet (S4) contained in-depth information about advantages, disadvantages and potential risks related to the BFR-LLST intervention. Additionally, any adverse event related to BFR-LLST at the supervised sessions or at home were reported by the patients to the physical therapist responsible for each BFR-LLST supervised session. If patients experienced clinical signs and symptoms of deep venous thrombosis at home, they were urged to contact a medical doctor or the physical therapist immediately (S4).

## 12. Setting

The exercises were performed at the supervised group-based classes twice a week at the Section for Orthopaedic and Sports Rehabilitation (SOS-R) Copenhagen, in the training hall. Home training were either performed in the patients’ own home or in a fitness center.

## 13.-15. Intervention description, Tailoring, Starting level

The usual care exercise after cartilage or meniscus repair in the knee joint - week 7 postoperatively – program contained 44 exercises (S6). The selection of exercises for each patient was based on their strength and quality of exercise performance.

Exercises were progressed if patients were able to perform the number of repetitions maximum (RM) and form/quality described in the usual care (exercise) template, starting from the lowest level. If the patients were able to complete all three sets with correct form and quality, the patients were progressed to the next exercise level for the specific muscle group. The patients did not progress, if the number of repetitions were not reached or if form/quality were not obtained as described.

If patients were not able to perform the next exercise as described in the template, because of pain or lack of strength, more resistance was added to the previous exercise, to ensure progression. The resistance applied in each exercise was registered and was used in the next training session at home.

Each BFR-LLST added to usual care exercise group-based session was initiated by ten minutes of pre-warming up exercises. Then 30 to 35 minutes were spent on exercises from the template, and the remaining time (10 to 15 minutes) was spent on BFR-LLST according to the protocol.

After the six weeks (week 7-12) with group-based usual care exercise supervised, the patients were instructed to perform the exercises three times per week. During the supervised rehabilitation phase, they were taught how to progress the exercises from the usual care exercise program.

The patients could experience acceptable pain during exercise. The pain needed to resolve immediately after performing the exercise (within 24 hours). The patients were instructed in differentiating between ‘good’ pain (muscle soreness/pain or pain around the knee) and ‘bad’ pain (pain inside the knee).

## 16. Fidelity to the intervention plan

At each BFR-LLST added to the usual care exercise session, number of training sessions performed for BFR-LLST (supervised and home), usual care exercise (supervised and at home), the BFR-LLST descriptors of limb occlusion pressure applied, number of sets, repetitions and external load lifted were recorded. BFR-LLST descriptors at home were patient-reported via a training diary (S4). Furthermore, the specific exercises performed at each usual care exercise supervised session were noted.

Adherence to the programs was acceptable with patients performing, on average, more than 80% of the scheduled BFR-LLST added to the usual care exercise sessions both supervised and at home were performed (S7 Table 2).
