# Supplementary material for: Blood flow restriction added to usual care exercise in patients with early weight bearing restrictions after cartilage or meniscus repair in the knee joint: a feasibility study
Source: J Exp Orthop. 2022 Oct 4;9:101. doi: 10.1186/s40634-022-00533-4 (PMC9530077; doi:10.1186/s40634-022-00533-4)
Supplement: Supplementary file 3 — Additional file 3: S3. Rehabilitation regimens - Cartilage (Steadman procedure) or meniscus repair. [file 40634_2022_533_MOESM3_ESM.docx]

| **Steadman procedure – Patellofemoral** | | | | |
| --- | --- | --- | --- | --- |
| **Weeks from** | **Bispebjerg Hospital** | **Exercise recommendation** | **Amager-Hvidovre Hospital** | **Exercise recommendation** |
| 0 | Full weight bearing on straight knee (knee extension 0 degrees)  No weight bearing on flexed knee | Passive and unloaded knee join range of motion (ROM) exercises 300 reps 3 times daily, e.g., on a stationary bike | Touchdown weight bearing from 0 to 20 degrees of knee joint flexion.  No weight bearing over 20 degrees of knee joint flexion. | Active and unloaded knee joint ROM exercises, 500 reps, 3 times daily. |
| 1 (after the 1^st^ week) | BFR-LLST knee-extension without load |  | BFR-LLST knee-extension without load |  |
| 3 (after the 2nd week) |  |  |  | Stationary bike unloaded |
| 7 (after the 6th week) | Progressed weight bearing as tolerated  Weight bearing to 90 degrees of knee joint flexion is allowed. |  |  |  |
| 7 (after the 6th week) | BFR-LLST knee-extension with external load |  |  |  |
| 9 (after the 8^th^ week) |  |  | BFR-LLST knee-extension with external load. Progressed weight bearing and gradually increase of knee joint flexion. |  |
| 13 (after 3 months) | Full weight bearing in the entire knee joint ROM |  |  |  |
| 17 (after 4 months) | Jogging/slow running  MD consultation |  | Progressive strength training  MD consultation |  |
| 27 (after 6 months) | Normal running and easy hopping/jumping activities on a flat surface. |  | Sports activities involving cutting, twisting, running and hopping. |  |

| **Steadman procedure – Tibiofemoral** | | | | |
| --- | --- | --- | --- | --- |
| **Weeks from** | **Bispebjerg Hospital** | **Exercise recommendation** | **Amager-Hvidovre Hospital** | **Exercise recommendation** |
| 0 | Touchdown weight bearing with use of crutches.  Full knee joint ROM. | Passive and unloaded range of motion (ROM) exercises 300 reps 3 times daily, e.g., on a stationary bike | Touchdown weight bearing with use of crutches.  Full knee joint ROM | Active and unloaded knee joint ROM exercises, 500 reps 3 times daily. |
| 1 (after the 1^st^ week) | BFR-LLST knee-extension without load |  | BFR-LLST knee-extension without load |  |
| 3 (after the 2nd week) |  |  |  | Stationary bike unloaded |
| 7 (after the 6th week) | Progressed weight bearing as tolerated.  Weight bearing until 90 degrees of knee joint flexion. |  | Progressed weight bearing as tolerated. |  |
| 13 (after 3 months) | Full weight bearing in the entire knee joint ROM |  |  |  |
| 17 (after 4 months) | Jogging/slow running |  | Progressive strength training  MD consultation |  |
| 27 (after 6 months) | Normal running and light hopping jumping activities on a flat surface  MD consultation |  |  | Sports activities involving cutting, twisting, running and hopping. |

| **Meniscus repair** | | | | |
| --- | --- | --- | --- | --- |
| **Weeks from** | **Bispebjerg Hospital** | **Exercise recommendation** | **Amager-Hvidovre Hospital** | **Exercise recommendation** |
| 0 | Immobilized in brace from -10 (extension below under 0 degree) to 40 degrees of knee joint flexion in 6 weeks.  Full weight bearing as tolerated in 0 degrees of knee joint flexion. No weight bearing on flexed knee joint. |  | Immobilized in brace. Full weight bearing as tolerated in 0 degrees of knee joint flexion.  No weight bearing on flexed knee joint. | Carefully unloaded active ROM exercises from 0 to 90 degrees of flexion as tolerated without brace. |
| 3 (after the 2nd week) | Supervised passive knee joint ROM from 0 to 90 degrees. | Carefully unloaded active ROM exercises from 0 to 90 degrees of flexion as tolerated without brace. | Brace with 0 to 90 degrees of flexion. No weight bearing over 90 degrees of flexion. |  |
| 3 (after the 2nd week) | BFR-LLST knee-extension without load |  | BFR-LLST knee-extension without load |  |
| 7 (after the 6th week) | Brace removal.  Full weight bearing as tolerated from 0 to 90 degrees flexion. Full ROM. | Full unloaded ROM exercises. | Brace removal.  Full weight bearing as tolerated from 0 to 90 degrees flexion. |  |
| 7 (after the 6th week) | BFR-LLST knee-extension with external load |  | BFR-LLST knee-extension with external load |  |
| 8 (after the 7th week) |  |  |  | Outside biking. |
| 13 (after the 3rd months) | MD consultation |  | Full weight bearing over 90 degrees of knee joint flexion. | Running on treadmill. |
| 17 (after the 4th months) | Full weight bearing over 90 degrees of knee joint flexion. |  | MD consultation. |  |
| 27 (after the 6th months) | Contact sport allowed. |  | Contact sport allowed. |  |

Range of motion = ROM; MD = Doctor of Medicine; Reps = repetitions.
