# Supplementary material for: Blood flow restriction added to usual care exercise in patients with early weight bearing restrictions after cartilage or meniscus repair in the knee joint: a feasibility study
Source: J Exp Orthop. 2022 Oct 4;9:101. doi: 10.1186/s40634-022-00533-4 (PMC9530077; doi:10.1186/s40634-022-00533-4)
Supplement: Supplementary file 4 — Additional file 4: S4. Blood Flow Restriction Exercise Leaflet. [file 40634_2022_533_MOESM4_ESM.pdf]

Blood flow restriction exercise

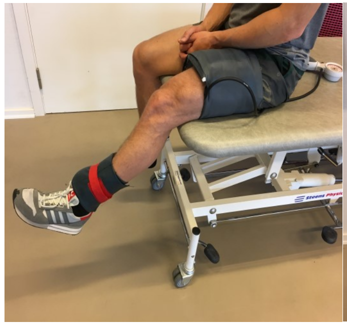
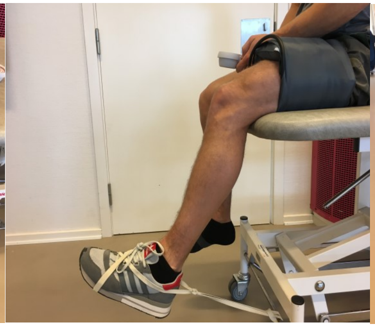
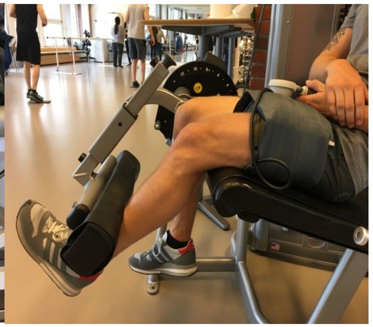
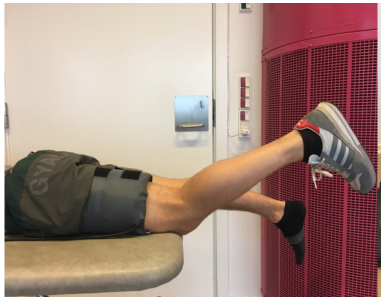
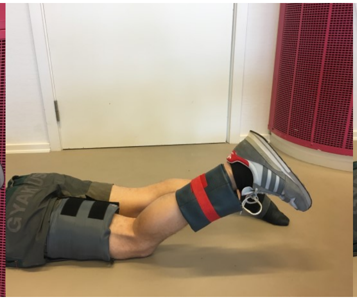
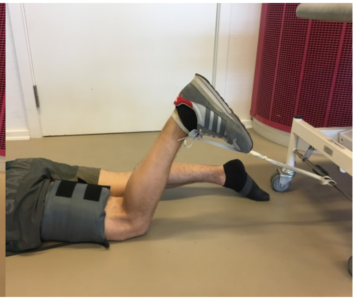

Prepared by the physical therapists Thomas Linding Jakobsen and Jakob Fisker, Section for Orthopedic and Sports Rehabilitation (SOS-R), Health Centre Nørrebro, City of Copenhagen

Patient:				Cuff width:				Limb occlusion pressure:			Physio
Week	Date	Cuff (C)/ Knee wrap (W)	Load (kg/elastic band (colour))	Set 1 Repetitions	Set 2 Repetitons	Set 3 Repetitions	Set 4 Repetitions	Muscle Pain	Knee Pain	Com-ments	Circumfe-rence (cm)
1											
2											
3											
4											
5											
6											

Blood flow restriction exercise (BFR)
Blow flow restriction exercise (BFR) is a form of training in which the blood flow is restricted (occluded) by an inflatable cuff or elastic band at the top of the thigh. This type of exercise has been found to increase muscle strength approximately to the same degree as traditional strength training. The advantage is that you can exercise intensively without high loads. When you are allowed to fully load your leg and experience acceptable knee pain, we recommend that you start traditional strength training.

Can I perform BFR exercise?
Standardized BFR exercise is safe. As we limit blood flow to the leg, we do not recommend BFR exercise if you:

- □ Have high blood pressure (hypertension), diabetes, and/or get bruising easily.
- □ Have problems with your heart or blood vessels (such as atherosclerosis).
- □ Have a neurological disease.
- □ Have a psychiatric diagnosis that prevents you from exercising regularly.
- □ Have an abuse of alcohol, medicine or euphoric drugs.
- □ Have cancer.
- □ Have an active inflammatory state in the body (septic active infection).
- □ Is pregnant.

BFR exercise in practice
The inflatable cuff is placed as far up on your thigh as possible. The physical therapist inflates the cuff to find the right pressure. Most often, the pressure will be between 80 and 120 mmHg (millimetres of mercury). A higher pressure may be used, if the pressure is determined by an oximeter or an ultrasound scanner with doppler function. The optimal pressure for you will be determined in collaboration with your physical therapist at your first visit.

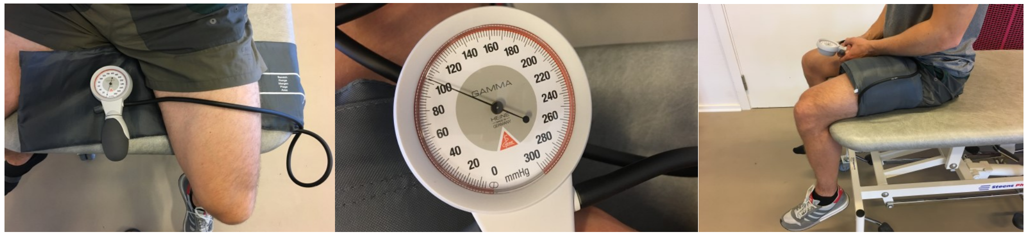

If you are using an elastic band, put the entire band around your thigh. It is placed in the same place as the cuff without space between the folds. You should experience the same pressure in your thigh as when using the cuff.

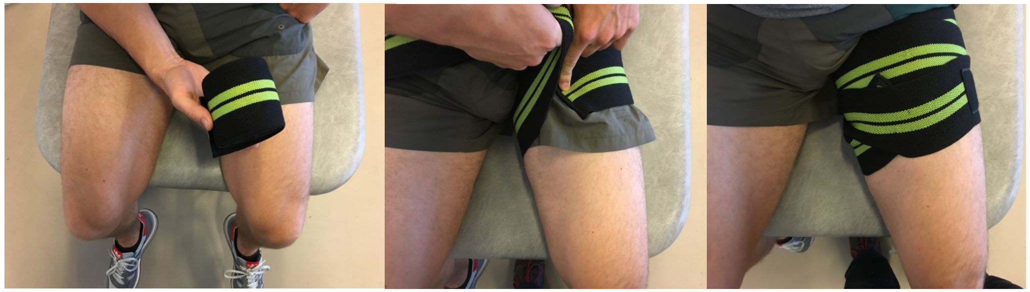

You have to complete 4 sets..
1. set: 30 repetitions, ½ minute break
2. set: 15 repetitions, ½ minute break
3. set: 15 repetitions, ½ minute break
4. set: 15 repetitions (Maximum 30 repetitions)

When performing a repetition, it should take 2 seconds to extend the leg to a horizontal position, 1 second to hold the position, 2 seconds to lower the leg again and 1 second to relax.
Do not restrict the thigh for more than 10 minutes while exercising.

<u>Load when performing BFR exercise:</u>
Start training without load. In the 4th and last set, perform as many repetitions as you can - however, no more than 30 repetitions. When you are allowed to load your knee, it is important that you gradually increase your exercise load. When you manage more than 2 repetitions on top of the 15 repetitions in your 4th and last set, the load should be increased in the next workout (+2 principle).

We recommend that you BFR exercise 5 times a week. It is an advantage to finish with the BFR exercise, when you train.

On the last page of the leaflet, there is a training diary, you are obliged to fill out. Then you and your physical therapist can follow your training. In this way we make sure that you train optimally.

If you want to train your posterior thigh muscle, the same principles apply as when you are training the anterior thigh muscle. The starting position is of course different. If you want to exercise both the anterior and posterior thigh muscles, there must be a 5-minute break, without occlusion around you thigh, in between your workouts.

Good advice
When you exercise, you will experience pain/discomfort in your muscle, but you must avoid pain in the knee joint. On a scale of 0 (no pain) to 10 (unbearable pain), avoid experiencing pain more than 3 out of 10 in the knee joint. You will sometimes notice that your leg becomes discoloured when performing BFR exercise. These symptoms are natural, and will disappear when you do not squeeze (occlude) your leg any more.

Once you have completed your BFR exercise, loosen the cuff/elastic band little by little. You may feel dizzy when you loosen your band. If you feel very dizzy, lie down with your legs elevated.

If you find that the leg is red all the time, it hurts, is swollen and you have a fever, you should contact a doctor or the responsible physical therapist immediately.

The physical therapist determines the pressure you need to work out with. The cuff or band you are training with should not squeeze more than a 7 on a scale from 0 (no pressure in the muscle) to 10 (unbearable pressure in the muscle).

Elastic bands (knee wraps) and inflatable cuffs are available in specialized stores. We recommend that you contact your physical therapist before purchasing.

It is advisable that the physical therapist determines your limb occlusion pressure (the pressure necessary to stop blood flow into your leg) with a oximeter or ultrasound scan-
