# Supplementary material for: Blood flow restriction added to usual care exercise in patients with early weight bearing restrictions after cartilage or meniscus repair in the knee joint: a feasibility study
Source: J Exp Orthop. 2022 Oct 4;9:101. doi: 10.1186/s40634-022-00533-4 (PMC9530077; doi:10.1186/s40634-022-00533-4)
Supplement: Supplementary file 5 — Additional file 5: S5. Usual care exercise after cartilage or meniscus repair in the knee joint - week 3–6 postoperatively. [file 40634_2022_533_MOESM5_ESM.docx]

# S5 REHABILITATION PROGRAM Cartilage repair procedure knee (tibiofemoral)

# (After 14 days and until week 6 incl.)

The exercise program is performed daily


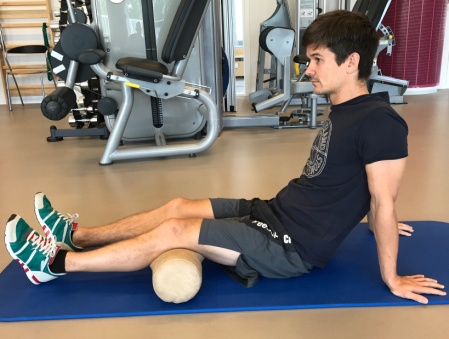

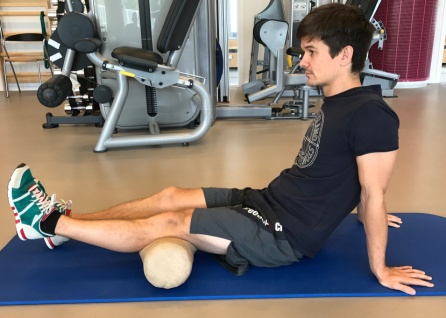


**1. Active knee extension**

Sit with extended leg on the floor or exercise mat.

Place a firm cushion under the knee.

Tighten up the front thigh muscle, so the heel is lifted from the floor to full knee extension. Hold the tension for 5 sec. then slowly lower the heel again.

**3 sets of 15 repetitions**

**2. Unloaded knee flexion**

Sit with the leg resting on a smooth surface and the foot placed on a piece of cloth. Then bend the knee, put your hands around the leg and pull your foot as far up towards the back of your thigh, while the foot slides on the surface. Then let the foot slide back in a controlled manner to the extended starting position.

**5 sets of 60 repetitions**

If you can flex more than 95 degrees in the knee, you replace the exercise with the exercise bike without resistance for 10 min.


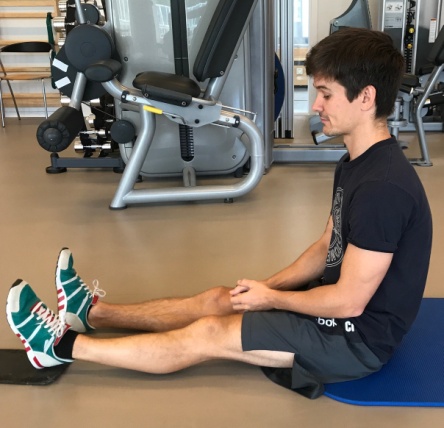

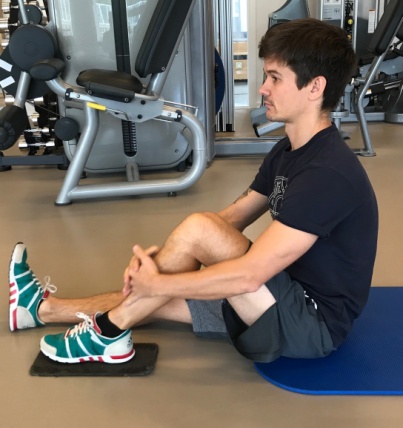


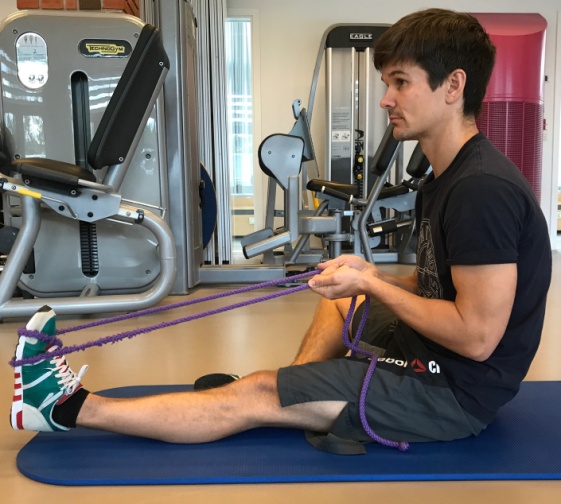


**3. Passive knee extension**

Sit with fully extended knee on the floor.

Extend the knee by the aid of a solid rope/strap, so that the heel is lifted from the surface and the knee is fully extended. Hold the extension for 5 sec., and then slowly lower the heel.

**3 sets of 15 repetitions**

**4. Prone knee flexion**

Lie in a prone position with fully extended knees. Bend the knee, so the heel is moved towards the buttock. Avoid swaying in the lower back, when the heel is pulled towards the buttock.

To increase the difficulty, put an elastic band around the ankles as resistance.

**3 sets of 15 repetitions**


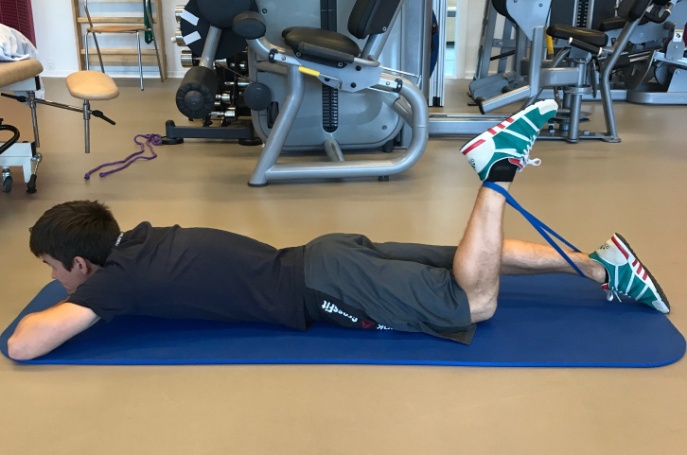


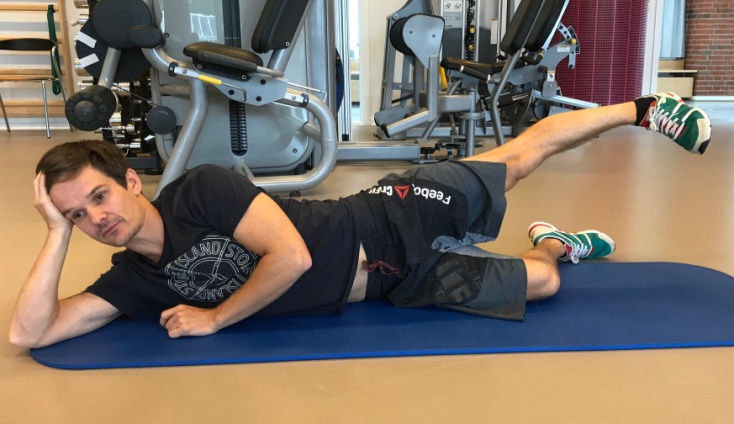


**5. Side laying stretched leg lift**

Lie on your side with the lower leg slightly bend to maintain optimal balance.

Lift the upper leg upwards and slanting backwards.

Slowly lower the leg again. It is important that you maintain a straight line between hip, knee and ankle during the entire movement.

**3 sets of 15 repetitions**

# S5 REHABILITATION PROGRAM Cartilage repair procedure knee (patellofemoral)

# (After 14 days and until week 6 incl.)

The exercise program is performed daily

**1. Active knee extension**

Sit with extended leg on the floor or mat. Place a firm cushion in the popliteal. Tighten up the front thigh muscle, so the heel is lifted from the floor to full knee extension.

Hold the tension for 5 sec. then slowly lower the heel again.

**3 sets of 15 repetitions**


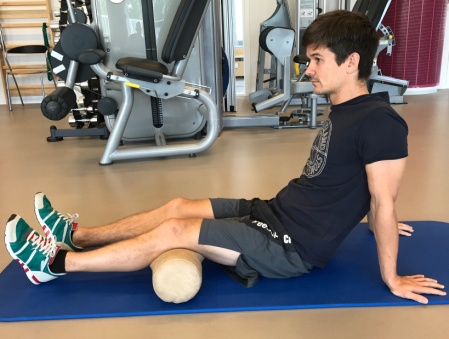

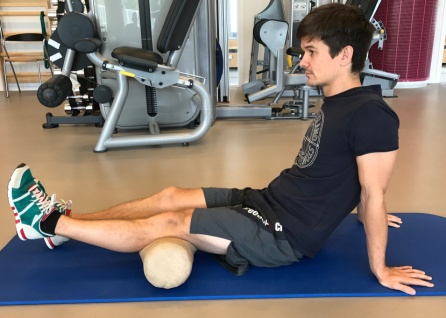


**2. Unloaded knee flexion**

Sit with the leg resting on a smooth surface and the foot placed on a piece of cloth. Then bend the knee, using the hands while the foot slides on the surface, then let the foot slide controlled back to extended starting position.

**5 sets of 60 repetitions**

If you can flex more than 95 degrees in the knee, you replace the exercise with the exercise bike without resistance for 10 min.


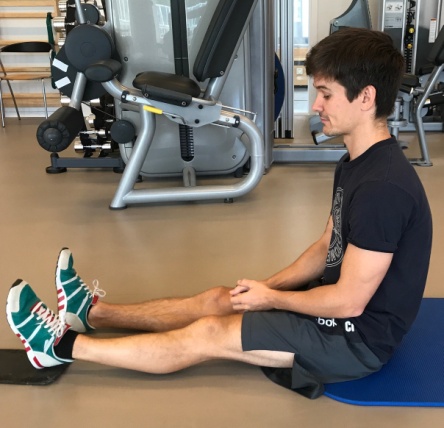

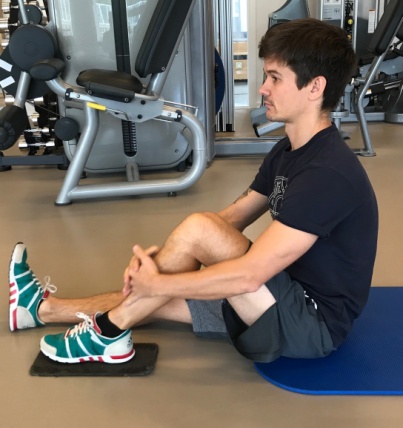


**3. Passive knee extension**

Sit with extended leg on the floor or other firm surface.

Extend the knee by the aid of a solid rope, so that the heel is lifted from the surface and the knee is fully extended. Hold the extension for 5 secs., and then slowly lower the heel.

**3 sets of 15 repetitions**


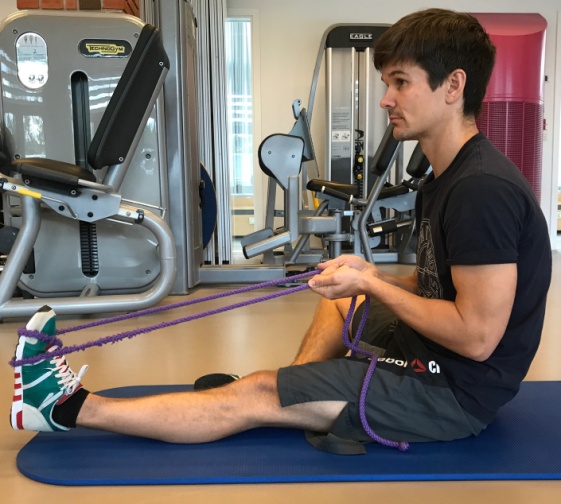


**4. Prone knee flexion**

Lie in a prone position with fully extended knees. Bend one knee, so the heel is moved towards the buttock. Avoid swaying in the lower back, when the heel is pulled towards the buttock.

To increase the difficulty, put an elastic band around the ankles as resistance.

**3 sets of 15 repetitions**


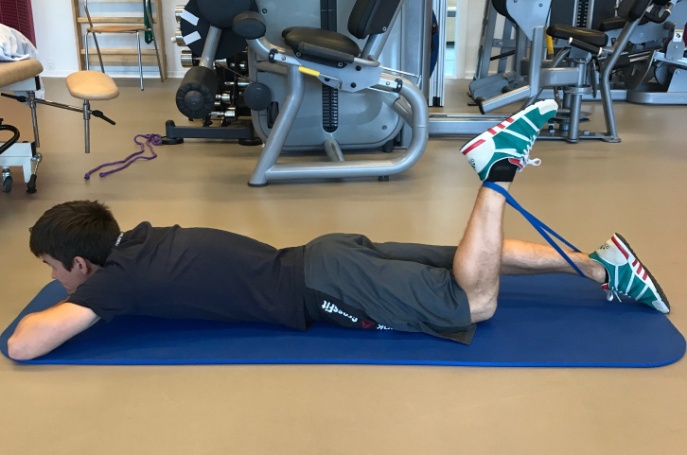


**5. Side laying stretched leg lift**

Lie on the side, with the lower leg slightly bend to maintain optimal balance.

Lift the upper leg upwards and slanting backwards.

Slowly lower the leg again. It is important that you maintain a straight line between hip, knee and ankle during the entire movement.

**3 sets of 15 repetitions**


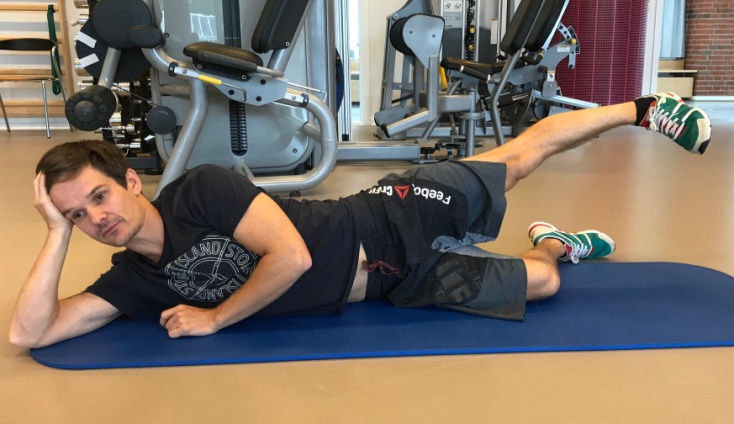


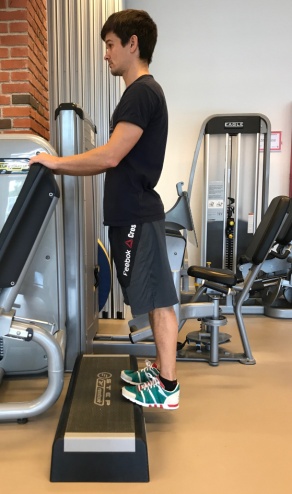

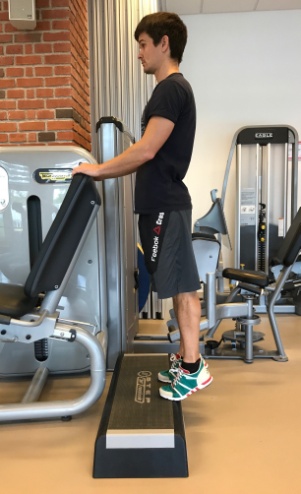


**6. Standing heel lift**

Stand on your forefoot with extended knees on a step with the heels slightly hanging off. Raise your body, so you are standing on your forefoot. Hold the position for 5 sec.

Then slowly lower the heels.

**3 sets of 15 repetitions**

# S5 REHABILITATION PROGRAM Meniscus repair

# (After 14 days and until week 6 incl.)

The exercise program is performed daily


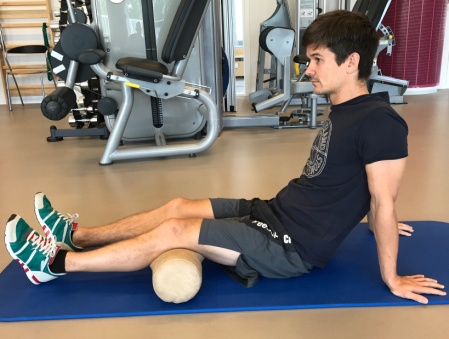

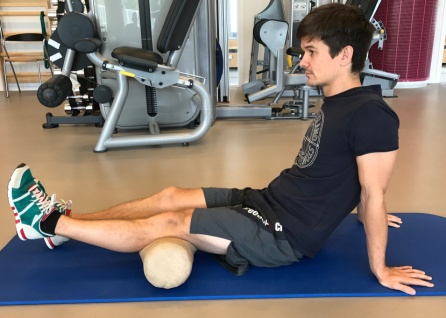


**1. Active knee extension**

Sit with extended leg on the floor or mat. Place a firm cushion in the popliteal. Tighten up the front thigh muscle, so the heel is lifted from the floor to full knee extension.

Hold the tension for 5 sec. then slowly lower the heel again.

**3 sets of 15 repetitions**


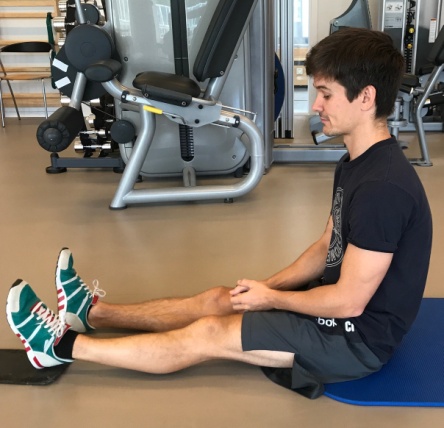

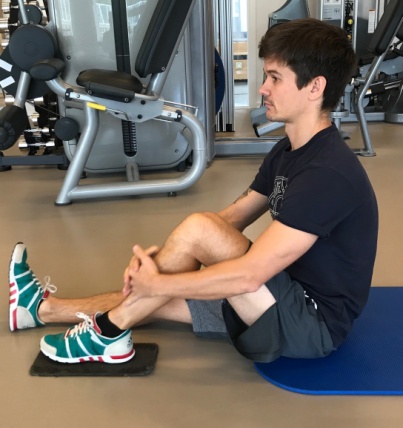


**2. Unloaded knee flexion**

Sit with the leg resting on a smooth surface and the foot placed on a piece of cloth. Then bend the knee, using the hands while the foot slides on the surface, then let the foot slide controlled back to extended starting position.

**Attention: Max. 90 degrees of flexion!**

**5 sets of 60 repititions**


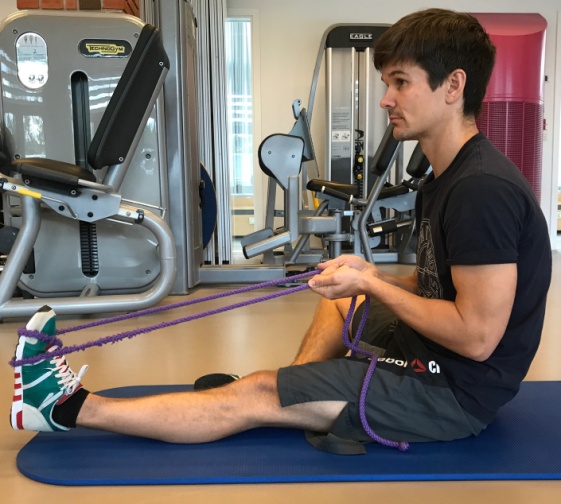


**3. Passive knee extension**

Sit with the leg on the floor or other firm surface. Extend the knee by the aid of a solid rope, so that the heel is lifted from the surface and the knee is fully extended. Hold the extension for 5 secs., and then slowly lower the heel.

**3 sets of 15 repetitions**


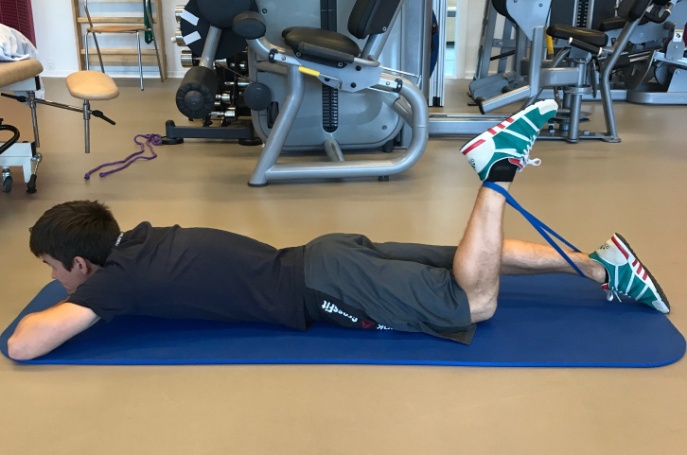


**4. Prone knee flexion**

Lie in a prone position with fully extended knees. Bend one knee, so the heel is moved towards the buttock. Avoid swaying in the lower back, when the heel is pulled towards the buttock.

To increase the difficulty, put an elastic band around the ankles as resistance.

**Attention: Max. 90 degrees of flexion!**

**3 sets of 15 repititions_**


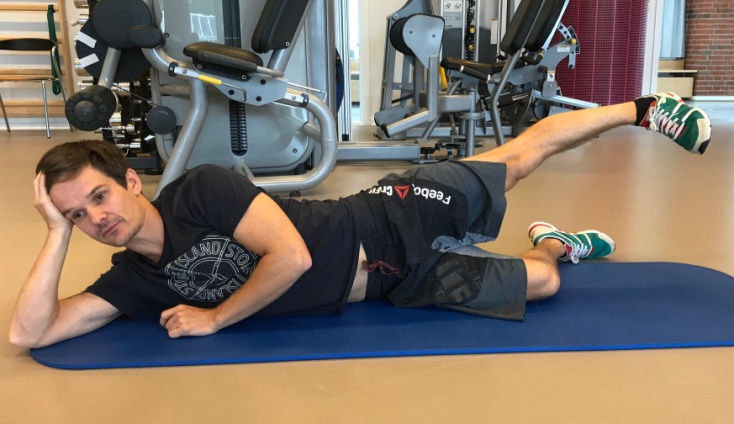


**5. Side laying stretched leg lift**

Lie on your side with the lower leg slightly bend to maintain optimal balance.

Lift the upper leg upwards and slanting backwards.

Slowly lower the leg again. It is important that you maintain a straight line between hip, knee and ankle during the entire movement.

**3 sets of 15 repetitions___**


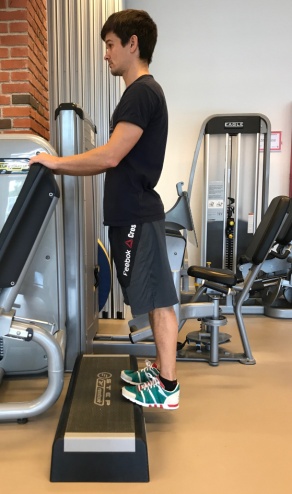

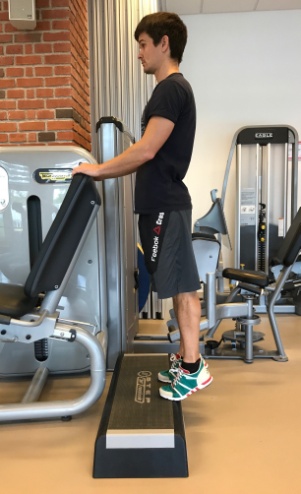


**6. Standing heel lift**

Stand with extended knees on the floor or a small step with the heels over the edge.

Raise the body, so you are standing on your toes. Hold the tension for 5 sec.

Then slowly lower the heels towards the floor.

**3 sets of 15 repititions**
