# Supplementary material for: Blood flow restriction added to usual care exercise in patients with early weight bearing restrictions after cartilage or meniscus repair in the knee joint: a feasibility study
Source: J Exp Orthop. 2022 Oct 4;9:101. doi: 10.1186/s40634-022-00533-4 (PMC9530077; doi:10.1186/s40634-022-00533-4)
Supplement: Supplementary file 6 — Additional file 6: S6. Usual care exercise after cartilage or meniscus repair in the knee joint - week 7 postoperatively. [file 40634_2022_533_MOESM6_ESM.pdf]

S6 Usual care exercise after cartilage or meniscus repair in the knee joint - week 7 postoperatively

Rehabilitation programme after cartilage or meniscus repair in the knee joint - week 7 postoperatively
The rehabilitation programme is developed by physiotherapists Jakob Fisker, Mikkel Hvidsteen and Thomas Linding Jakobsen, Section for Orthopedic and Sports Rehabilitation, (SOS-R), Centre of Rehabilitation - Nørrebro, City of Copenhagen, Denmark.
Your treating physiotherapist will choose the exercises, you have to perform. When you are allowed to perform the described exercises depends on the restrictions given by the hospital where you had your operation.
Perform the exercises two times per week with a physiotherapist at Centre of Rehabilitation - Nørrebro and once a week in a gym. You have to do the same exercises in the gym, as you do with your physiotherapist.
Perform the BFR training five times per week - twice at the Centre of Rehabilitation - Nørrebro and three times at home or in the gym.

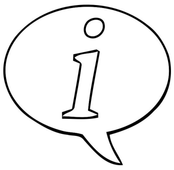
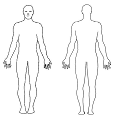

1. Warming up

Warming up – before your supervised group-based training you must either cycle or use a rowing machine. This depends on the instructions given by your physiotherapist.

Duration: 10 Min

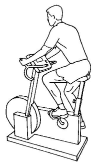
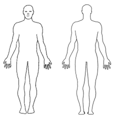

2. Bike

You can choose a program and start the bike, or press quick start. Press plus/minus to change resistance. To finish press stop.

Anstrengelsesgrad: 14-17 Borg skala

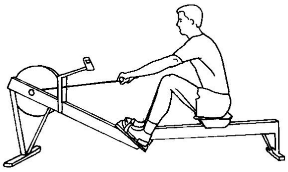
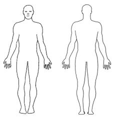

3. Rowing machine

Adjust resistance by using the handle to your right. Press "on", choose the information you wish to see on the screen and start rowing. Pull the handle towards your abdomen while pushing with your legs until they are straight.

Angstrengelsesgrad: 14-17 Borg skala

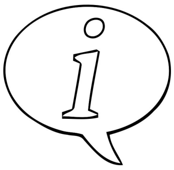
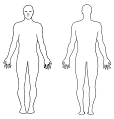

4. Warming up in the training room

Spend 5 minutes on carpet tile exercises and /or walking lunge without/with weights.
Thereafter, spend 5 minutes on the sideways walk with elastic.
Duration: 10 minutes in total

Duration: 10 Min

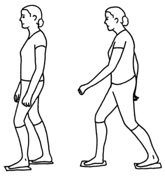
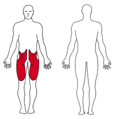

5. Slides: Sliding walk

Place two pieces of carpet tile under your feet. Walk and slide at the same time and place your body weight alternately on the right and left leg.

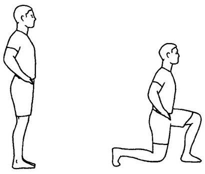
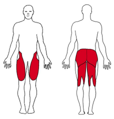

6. Forward lunge

Stand with your legs together and place your hands on your hips. Lift one leg and take a step forward. When your leg touches the floor, slow down the movement until your body is in the bottom position. Keep your upper body straight. Press back and take a new step forward. Keeping control of your knees and maintaining a neutral back is important throughout the entire movement.

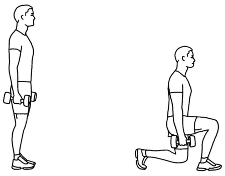
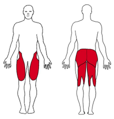

7. Walking lunges with dumbbells

Stand with your legs together and a dumbbell in each hand. Lift one leg, and then walk forward in a straight line. When the back leg hits the floor push off and bring it forward, take a new step with the other e.g. The upper-body should be straight. Keep the knees above the toes throughout the entire exercise.

Show video

S6 Usual care exercise after cartilage or meniscus repair in the knee joint - week 7 postoperatively

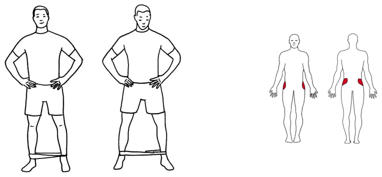

8. Mini Band Side Steps
Stand upright on the floor with feet positioned at hip width and an elastic band around your ankles. Walk sideways, preferably with your feet rotated slightly inwards. Keep tension in the band during the exercise.

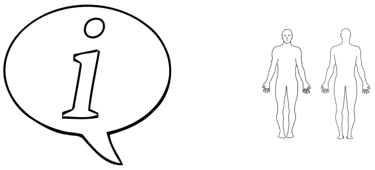

9. Strength exercises for the hip abductor muscles
The exercise is performed to fatigue. That is, when you can perform more repetitions than 12 with good technique in each set, the exercise must be made more difficult.

You increase the load with more weight / heavier elastic or you switch to a more demanding exercise. Each repetition lasts 4 seconds, where it takes 2 seconds to lift (concentric) and 2 seconds to lower (eccentric) the weight. The pause between each set should be between 1 and 5 minutes.
The strength exercise is performed only for the operated leg, if it is a 1-leg exercise.

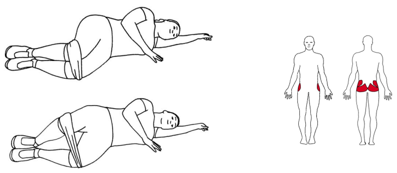

10. The clam w/ elastics
Lay on your side on a mat with the lower arm straight, resting your head on your arm. Bend your legs and place an elastic band around your knees. While the lower leg is held still, lift the upper leg up toward the ceiling, against the resistance of the elastic band. Keep your upper hip pointing towards the floor

Sets: 3 , Reps: 12 , Duration: 4 sec

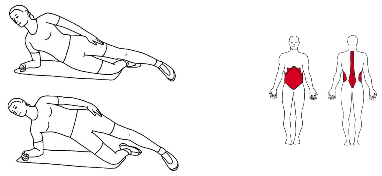

11. Side Plank on Knees with Leg Raises
Lie on your side with the lower leg bent and support yourself by using your elbow. Tighten up your abdominals and raise your pelvis from the surface until your body forms a straight line. Then, raise the upper leg towards the ceiling and lower it steadily again.

Sets: 3 , Reps: 12 , Duration: 4 sec

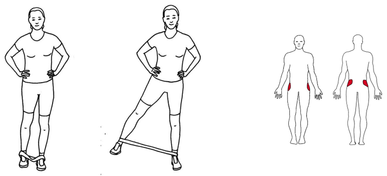

12. Abduction of the hip with elastic band
Secure the strap around the ankles. Stretch one leg out to the side with your toes pointing forward. Slowly go back to the starting position and repeat. Keep your upper body still during the movement.

Sets: 3 , Reps: 12 , Duration: 4 sec

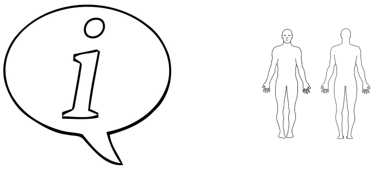

13. Strength exercises of the hamstring and buttock muscles
The exercise is performed to fatigue. That is, when you can perform more repetitions than 12 in your good technique set, the exercise must be made more difficult.

You increase the load with more weight / elastic with higher resistance or you switch to a more demanding exercise. Each repetition lasts 4 seconds, where it takes 2 seconds to lift (concentric) and 2 seconds to lower (eccentric) the weight. The pause between each set should be between 1 and 5 minutes. The strength exercise is performed only for the operated leg, if it is a 1-leg exercise.

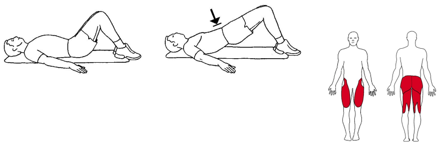

14. "Bridge"
Lie on your back with the soles of your feet on the floor and your arms out to the side. Tilt your pelvis backward to neutral position.
Activate the lower part of your abdominal muscles, pull your navel inward and "flatten" your abdomen. Raise your pelvis from the floor until you lie on your shoulders.

Sets: 3 , Reps: 12 , Duration: 4 sec

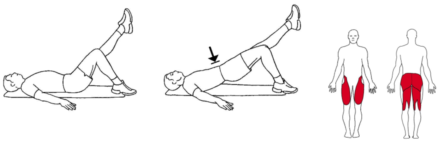

15. Single Leg Glute Bridge, side view
Lie on your back with the soles of your feet on the floor and your arms out to the side. Tilt your pelvis backward. Activate the lower part of your abdominal muscles, pull your navel inward and "flatten" your abdomen. Carefully straighten one leg and raise your pelvis from the floor until you lie on your shoulders. Repeat with your other leg.

Sets: 3 , Reps: 12 , Duration: 4 sec

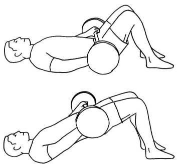
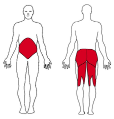

16. Barbell Glute Bridge

Lie on your back with legs bent and hold a barbell over your hips. Flex your abs and raise your buttocks from the floor until there is a straight line from shoulders to knees. Hold a few seconds and slowly lower to starting position.

Sets: 3 , Reps: 12 , Duration: 4 sec

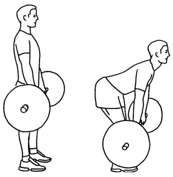
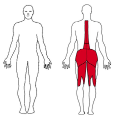

17. Romanian Deadlift 2

Hold the bar with a overhand grip hanging in front of you at hip level. Shoulders should be slightly retracted, chest pushed out, the back naturally arched and the knees slightly bent. Lower the bar by moving your buttocks backwards and lowering the upper body forwards. Keep looking forward, the shoulders retracted and the spine straight during the movement. Lower the bar directly downwards, close to your body. Go as far as muscle flexibility allows and return to the starting position by driving the hips forwards.

Sets: 3 , Reps: 12 , Duration: 4 sec

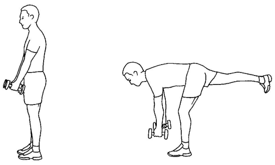
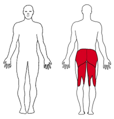

18. Single Leg Romanian Dumbbell Deadlift

Stand on one leg with a weight in each hand. Keep your back straight and look ahead. Lift one leg beackwards while you gradually bend your upper body forward. Sligthly bend the knee on the leg that you are standing on. Keep your back straight as you bend over. The entire movement is made in your hip joint. Move your upper body back up while you lower your leg back to starting position.

Sets: 3 , Reps: 12 , Duration: 4 sec

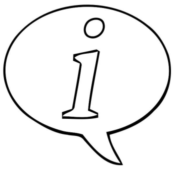
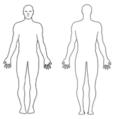

19. Strength exercises for the hip adductor muscles

The exercise is performed to fatigue. That is, when you can perform more repetitions than 12 in your good technique set, the exercise must be made more difficult.

You increase the load with more weight / elastic with higher resistance or you switch to a more demanding exercise. Each repetition lasts 4 seconds, where it takes 2 seconds to lift (concentric) and 2 seconds to lower (eccentric) the weight. The pause between each set should be between 1 and 5 minutes. The strength exercise is performed only for the operated leg, if it is a 1-leg exercise.

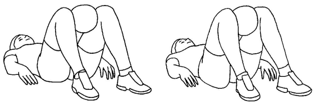
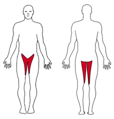

20. On your back "squeeze" of ball 1

Lie on your back with your knees bend and the soles of your feet placed on the ground. Place a football or similar ball between your knees.
Press your knees together, in a way where you squeeze the football as flat as possible. Easily release again.

Sets: 3 , Reps: 12 , Duration: 4 sec

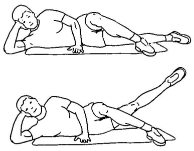
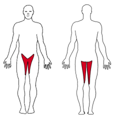

21. Side-lying single leg lift 2

Lie on your side, supporting your head with one hand. Cross your upper leg in front of the other. Raise your lower leg toward the ceiling. Slowly return to the start position and repeat. Change legs.

Sets: 3 , Reps: 12 , Duration: 4 sec

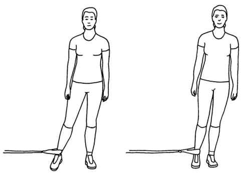
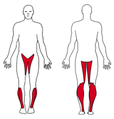

22. Standing inward leg pull w/elastic band 2

Stand with your side facing the point of attachment. Secure the elastic band at ankle height and attach it around your ankle. You should start the exercise with your active leg slightly abducted. Slowly move your leg towards the midline and return to starting position. The foot is facing forward throughout the exercise.

Sets: 3 , Reps: 12 , Duration: 4 sec

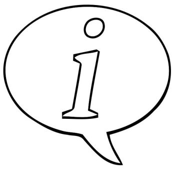
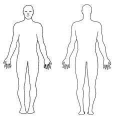

23. Strength training of the calf muscles

The exercise is performed to fatigue.

That is, when you can perform more repetitions than 12 with good technique in each set, the exercise is made more difficult.

You increase the load with more weight / heavier elastic or you switch to a more demanding exercise. Each repetition lasts 4 seconds, 2 seconds to lift (concentric) and 2 seconds to lower (eccentric) the weight. The pause between each set should be between 1 and 5 minutes. The strength exercise is performed only for the operated leg, if it is a 1-leg exercise

S6 Usual care exercise after cartilage or meniscus repair in the knee joint - week 7 postoperatively

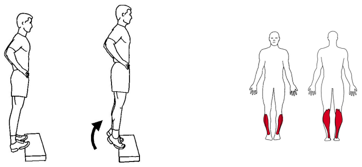

24. Elevated Calf Raises

Stand on the edge of a step. The stairs or a bench may be used as well. Let your heels hang over the edge, feet about hip-width apart. Raise your heels and push up until you are on your toes. Return to the starting position and repeat. The exercise can be done with or without support.

Sets: 3 , Reps: 12 , Duration: 4 sec

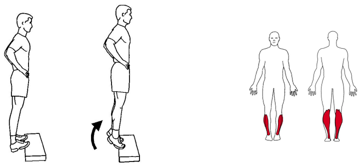

25. Elevated Calf Raises w/ dumbbells

Hold a dumbbell in each hand. Stand on the edge of a step. The stairs or a bench may be used as well. Let your heels hang free, feet about hip-width apart. Raise your heels and push up until you are on your toes. Return to the starting position and repeat. The exercise can be done with one or 2 dumbbells.

Sets: 3 , Reps: 12 , Duration: 4 sec

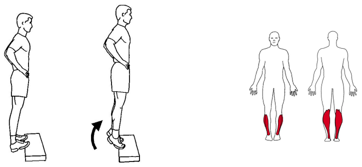

26. Elevated Calf Raises - One-legged

Stand on one leg the edge of a step. The stairs or a bench may be used as well. Let your heel hang free. Raise your heel and push up until you are on your toe. Return to the starting position and repeat. The exercise can be done with or without support.

Sets: 3 , Reps: 12 , Duration: 4 sec

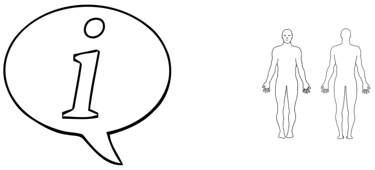

27. Strength training of the quadriceps muscles

The exercise is performed to fatigue.

That is, when you can perform more repetitions than 12 with good technique in each set, the exercise is made more difficult.

You increase the load with more weight / heavier elastic or you switch to a more demanding exercise. Each repetition lasts 4 seconds, 2 seconds to lift (concentric) and 2 seconds to lower (eccentric) the weight. The pause between each set should be between 1 and 5 minutes. The strength exercise is performed only for the operated leg, if it is a 1-leg exercise

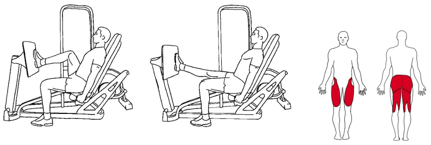

28. Cybex: Seated Leg Press 2

Put one foot on the platform. Ensure that your knee is bent 90 degrees. Contract your abdominal and lower back muscles and push your leg forward. Stop the movement when your leg is almost straight to avoid overstretching your knee. Lower your leg toward you at a slightly slower pace. Repeat the exercise with your opposite leg.

Sets: 3 , Reps: 12 , Duration: 4 sec

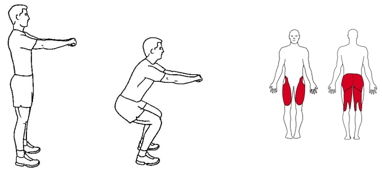

29. Squat 1

Stand with your feet shoulder-width apart and arms straight in front of you. Bend your knees 90 degrees and push back up. Keep your back straight and look straight ahead during the entire movement. Alternatively, the deep position may be held for a few seconds before pushing back up.

Sets: 3 , Reps: 12 , Duration: 4 sec

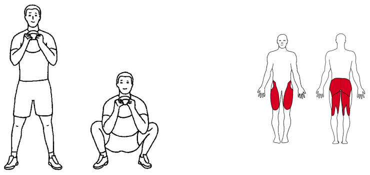

30. Goblet Squat

Stand with your legs wide apart with a kettlebell in your hands in front of your chest. Move your body downwards and do a deep knee bend. Be aware that your knees and toes point in the same direction. Move back up to starting position again.

Sets: 3 , Reps: 12 , Duration: 4 sec

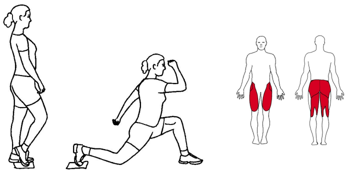

31. Slides: Back Lunge

Focus on a spot in front of you. Tighten your abdomen, place one foot on a cloth and move backwards. Lower your back knee down towards the floor. Press your bottom down towards the floor, tighten abdomen and move your leg back to starting position.

Sets: 3 , Reps: 12 , Duration: 4 sec

Show video

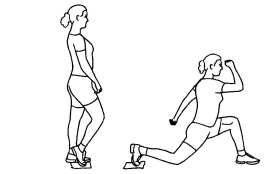
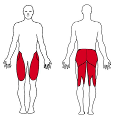

32. Slides: Back Lunge w/ dumbbells
Hold a dumbbell in each hand. Focus on a spot in front of you. Tighten your abdomen, place one foot on a cloth and move backwards. Lower your back knee down towards the floor. Press your bottom down towards the floor, tighten abdomen and move your leg back to starting position.
Sets: 3 , Reps: 12 , Duration: 4 sec

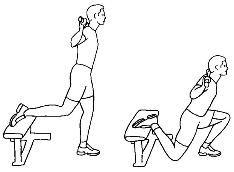
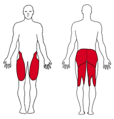

33. Bulgarian Split Squat w/barbell
Look straight during the entire exercise. Start on one leg. Distribute most of your weight to your front foot. The other leg should rest on a bench and be passive during the whole exercise. Keep your body still during the entire exercise. Breathe in on your way down, hold your breath at the lowest part of the movement and breathe out on your way back up. Finish the movement at 90 degrees angle in your knee.
Sets: 3 , Reps: 12 , Duration: 4 sec

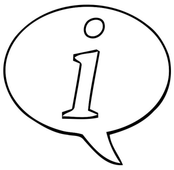
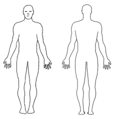

34. Balance and stability exercises
The balance exercises are performed on one or two legs on different surfaces. It can be an Airex pillow, rocker or BOSU ball. In addition, balls or other can be used in the individual balance exercises.
Duration: 5 Min

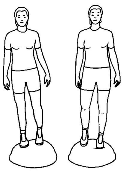
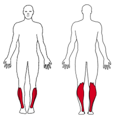

35. BOSU-ball: balance 1
Stand with the feet parallel slightly apart on the top of the BOSU-ball. Move the weight in front of the toes, back on the heels, and sideways. You can perform the exercise with closed eyes to increase the degree of difficulty.

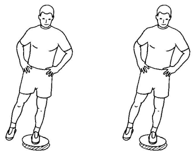
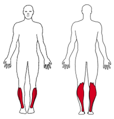

36. Balance board: single leg balance 1
Stand on one leg on the balance board and try to keep your balance. The degree of difficulty increases if you move the other leg. Change legs.

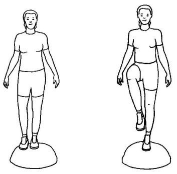
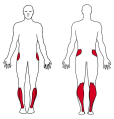

37. BOSU-ball: Balance 3
Stand with one leg on the BOSU-ball: Lift the other leg. Hold the position for a few seconds before you put the foot down again. Repeat for each one at a time.

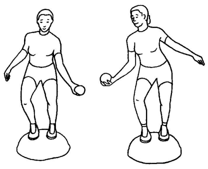
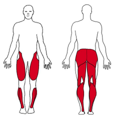

38. BOSU-ball: Balance w/ weightball 2
Stand with the feet paralle and feet slightly apart on the top of the BOSU-ball. Bend the knees and hips. Make sure that you keep the back straight and the neck in a neutral extension of the back. Throw the ball from one hand to the other. Straighten yourself up and return to the starting position. Repeat to the other side. Increase the degree of difficulty by letting the eyes follow the ball, throw high, throw the ball further to the side and increase the tempo.

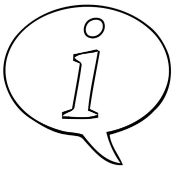
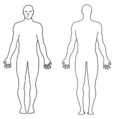

39. Blood flow restriction - Low load strength training
Please follow the handed out leaflet
Duration: 10 Min

Show video

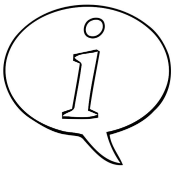
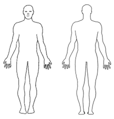

40. Stretching exercises

When stretching your muscles, feel a stretch in the muscles you want to stretch. You need to keep stretching your muscles for 30 seconds. It is an advantage that you breathe deeply and calmly as you stretch the muscle.

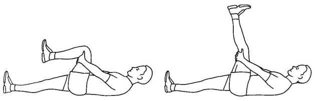
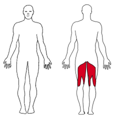

41. The Extender

In supine position. Bend one of your legs and grab hold of the back of your knee. Maintain a 90 degree angle in your hip while extending your knee. Extend and stretch the back side of your leg. Stop just before pain is felt.

Duration: 30 sec, Reps: 2

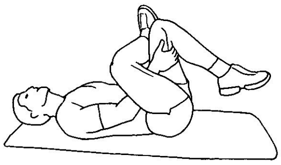
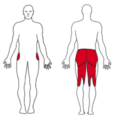

42. Buttocks 2

Lie on your back with one leg crossed over the other so that your ankle rests on the opposite knee. Hold your thigh and pull it toward your chest until you feel your buttocks muscles stretch. Hold for 30 seconds and change legs.

Duration: 30 sec, Reps: 2

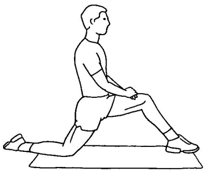
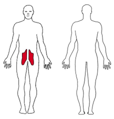

43. Hip flexors 1

Place one knee on the floor and the other leg's foot in front of you. Place both hands on your thigh for support and slowly push your hip forward into the stretch keeping your upper body upright. Hold for 30 seconds and repeat with your other leg.

Duration: 30 sec, Reps: 2

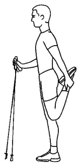
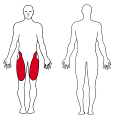

44. Stretching of the front thigh 1

Stand with the body straight and support yourself with the rods. Grab one of the ankle, and pull the heel towards the back. Push the pelvic forward, until you feel the front of the thigh stretching. Hold the position for 30 seconds. Repeat with the other leg.

Duration: 30 sec, Reps: 2

Show video
