# Supplementary material for: Blood flow restriction added to usual care exercise in patients with early weight bearing restrictions after cartilage or meniscus repair in the knee joint: a feasibility study
Source: J Exp Orthop. 2022 Oct 4;9:101. doi: 10.1186/s40634-022-00533-4 (PMC9530077; doi:10.1186/s40634-022-00533-4)
Supplement: Supplementary file 7 — Additional file 7: S7. Supplementary results. S7 Table 1. Patient characteristics at 16 and 26-week assessment. S7 Table 2. Clinical application (adherence), training characteristics and pain at rest and during BFR-LLST added to usual care exercise. S7 Table 3. Change per week in thigh circumference, knee joint and quadriceps pain, perceived exertion and training load during the, on average, 11 weeks of BFR-LLST added to usual care exercise intervention period (15 sessions). S7 Table 4. Number of exercises performed during the group-based usual care exercise supervised program (15 sessions). S7 Table 5. Adverse events during the BFR-LLST added to usual care exercise intervention period. S7 Table 6. Average and overall change in knee joint pain, quadriceps muscle pain and perceived exertion from 1st to 4th set within the BFR-LLST session (15 sessions) for all patients (n = 42). [file 40634_2022_533_MOESM7_ESM.docx]

S7 Supplementary Results

Content

[S7 Table 1 Patient characteristics at 16 and 26-week assessment 2](#_Toc88828693)

[S7 Table 2 Clinical application (adherence)., training characteristics and pain at rest and during BFR-LLST added to usual care exercise 3](#_Toc88828694)

[S7 Table 3 Change per week in thigh circumference, knee joint and quadriceps pain, perceived exertion and training load during the, on average, 11 weeks of BFR-LLST added to usual care exercise intervention period (15 sessions). 4](#_Toc88828695)

[S7 Table 4 Number of exercises performed during the group-based usual care exercise supervised program (15 sessions) 5](#_Toc88828696)

[S7 Table 5 Adverse events during the BFR-LLST added to usual care exercise intervention period 6](#_Toc88828697)

[S7 Table 6 Average and overall change in knee joint pain, quadriceps muscle pain and perceived exertion from 1^st^ to 4^th^ set within the BFR-LLST session (15 sessions) for all patients (n=42) 7](#_Toc88828698)

## S7 Table 1 Patient characteristics at 16 and 26-week assessment

| Patient characteristics |  |  |
| --- | --- | --- |
| Assessment 16 weeks postoperatively | Cartilage (n=21) | Meniscus (n=21) |
| Continue doing BFR exercise, no (%) | 15 (71) | 10 (48) |
| Cuff (knee wraps/inflatable cuff/both), no (%) | 8 (53)/ 0 (0)/ 7 (47) | 3 (10)/ 1 (10)/ 6 (60) |
| Number of exercises per week, median (IQR), no | 5 (3 to 5) | 3 (2 to 5) |
| Postoperative complications, no (%) | 4 (19)^a^ | 3 (14)^b^ |
| Pain medication before assessment, no (%) | 0 (0) | 0 (0) |
| Acetaminophen (Paracetamol), no (%) | 0 (0) | 0 (0) |
| NSAID, no (%) | 0 (0) | 0 (0) |
| Opioids and opioid-like drugs, no (%) | 0 (0) | 0 (0) |
|  | | |
| Assessment 26 weeks postoperatively | Cartilage (n=20) | Meniscus (n=19) |
| Doing BFR exercise, no (%) | 5 (25) | 4 (26) |
| Cuff (knee wraps/inflatable cuff/both), no (%) | 5 (25)/0 (0)/0 (0) | 4 (26)/0 (0)/0 (0) |
| Number of exercises per week, median (IQR), no | 3 (3 to 4) | 3 (2.5 to 3.0) |
| Postoperative complications, no (%) | 5 (25)^c^ | 8 (42)^d^ |
| Pain medication before assessment, no (%) | 0 (0) | 1 (5) |
| Acetaminophen (Paracetamol), no (%) | 0 (0) | 1 (5) |
| NSAID, no (%) | 0 (0) | 1 (5) |
| Opioids and opioid-like drugs, no (%) | 0 (0) | 0 (0) |

Abbreviations: BFR; Blood flow restriction. IQR; Interquartile Range. NSAID; Non-Steroidal Anti-Inflammatory Drug.

^a^Knee joint pain (n=3), Knee swelling (n=1). ^b^Knee joint pain (n=2), Tibial fixation screw pain (n=1). ^c^Knee joint pain (n=5), ^d^Knee joint pain (n=3), Knee swelling (n=1), Sensation of something inside the knee (n=1), Varicosa veins (n=1), Knee instability (n=1), Meniscal resection opposite knee (n=1).

## S7 Table 2 Clinical application (adherence), training characteristics and pain at rest and during BFR-LLST added to usual care exercise

|  | **Cartilage (n=21)** | **Meniscus (n=21)** | **Total (n=42)** |
| --- | --- | --- | --- |
| **Clinical application (adherence)^a^** | | | |
| BFR-LLST supervised, mean 1±SD (range, % completed), sessions | 13 ± 1 (9-15, 84) | 13 ± 2 (9-15, 84) | 13 ± 2 (9-15, 84) |
| BFR-LLST home, mean 1±SD (range, % completed), sessions | 37 ± 9 (21-57, 124) | 34 ± 5 (26-43, 113) | 35 ± 8 (21-57, 118) |
| BFR-LLST total, mean 1±SD (range, % completed), sessions | 50 ± 10 (34-70, 110) | 47 ± 6 (36-58, 107) | 48 ± 8 (31-70, 107) |
| Usual care exercise supervised, mean 1±SD (range, % completed), sessions | 13 ± 1 (9-15, 84) | 13 ± 2 (9-15, 84) | 13 ± 2 (9-15, 84) |
| Usual care exercise home, mean 1±SD (range, % completed), sessions | 31 ± 13 (14-61, 94) | 29 ± 12 (1-48, 97) | 30 ± 12 (1-61, 102) |
| Usual care exercise total, mean 1±SD (range, % completed), sessions | 43 ± 12 (26-74, 92) | 42 ± 12 (13-63, 94) | 42 ± 12 (13-74, 98) |
| **Training characteristics** | | | |
| Limb occlusion pressure (LOP), mean ±1SD (range), mmHg^b^ | 188 ± 17 (160-240) | 187 ± 22 (150-240) | 187 ± 19 (150-240) |
| Relative applied 80% limb occlusion pressure (LOP), mean ±1SD (range), mmHg^c^ | 135 ± 14 (112-176) | 134 ± 17 (104-176) | 134 ± 15 (104-176) |
| Number of repetitions per BFR-LLST session, mean ±1SD (range), no | 82 ± 12 (15-136) | 81 ±12 (10-135) | 81 ± 12 (10-136) |
| Performed the recommended number of repetitions per BFR-LLST session (min. 75 repetitions), mean ± 1SD, % | 80 ± 4 | 78 ± 4 | 79 ± 4 |
| External training load during BFR, median (IQR, range), kg | 1 (0 to 2, 0-7) | 1 (0 to 2, 0-5) | 1 (0 to 2, 0-7) |
| **Knee joint and quadriceps pain** | | | |
| Knee joint pain at rest before BFR-LLST supervised session, median (IQR, range), VAS-mm | 0 (0 to 0, 0-61) | 0 (0 to 1, 0-70) | 0 (0 to 0, 0-70) |
| Knee joint pain at rest after BFR-LLST supervised session, median (IQR, range), VAS-mm | 0 (0 to 0, 0-65) | 0 (0 to 0, 0-49) | 0 (0 to 0, 0-65) |
| Quadriceps muscle pain at rest before BFR-LLST supervised session, median (IQR, range), VAS-mm | 0 (0 to 0, 0-51) | 0 (0 to 0, 0-40) | 0 (0 to 0, 0-51) |
| Quadriceps muscle pain at rest after BFR-LLST supervised session, median (IQR, range), VAS-mm | 0 (0 to 13, 0-70) | 0 (0 to 9, 0-49) | 0 (0 to 10, 0-70) |
| Maximal knee joint pain experienced during usual care exercise supervised, median (IQR, range), VAS-mm | 21 (0 to 35, 0-100) | 25 (0 to 42, 0-88) | 23 (0 to 40, 0-100) |
| Maximal quadriceps muscle pain experienced during usual care exercise supervised, median (IQR, range), VAS-mm | 0 (0 to 7, 0-80) | 0 (0 to 5, 0-65) | 0 (0 to 6, 0-80) |
| Maximal knee joint pain experienced during BFR-LLST supervised session, median (IQR, range), VAS-mm | 0 (0 to 10, 0-93) | 0 (0 to 16, 0-92) | 0 (0 to 12, 0-93) |
| Maximal quadriceps muscle pain experienced during BFR-LLST supervised session, median (IQR, range), VAS-mm | 52 (15 to 71, 0-100) | 40 (0 to 68, 0-100) | 47 (12 to 70, 0-100) |

Abbreviations: BFR-LLST; Blood flow restriction – low load strength training. SD; Standard Deviation. IQR; Interquartile Range. VAS; Visual Analog Scale.

^a^Total number of exercise (BFR/Usual care exercise supervised (n=15), BFR home (n=30), Usual care exercise home (n=48), BFR total (n=45), Usual care exercise total (n=63)). ^b^Two patients (meniscus repair (n=1), cartilage repair (n=1)) lowered their limb occlusion pressure applied due to discomfort; one transient and one permanent. ^c^Relative applied 80% LOP: LOP minus 20 mmHg (variability) multiplied with 0.8.

## S7 Table 3 Change per week in thigh circumference, knee joint and quadriceps pain, perceived exertion and training load during the, on average, 11 weeks of BFR-LLST added to usual care exercise intervention period (15 sessions).

| Outcome measure | Change per week | | | | | |
| --- | --- | --- | --- | --- | --- | --- |
|  | Cartilage (n=21) | | Meniscus (n=21) | | All (n=42) | |
|  | mean (95% CI) | p-value | mean (95% CI) | p-value | mean (95% CI) | p-value |
| Thigh circumference - operated leg, cm | 0.05 (-0.01 to 0.11) | <0.099 | 0.12 (0.07 to 0.17) | <0.001 | 0.09 (0.05 to 0.13) | <0.001 |
| Thigh circumference - healthy leg, cm | -0.01 (-0.05 to 0.04) | <0.801 | 0.02 (-0.03 to 0.07) | <0.425 | 0.01 (-0.03 to 0.04) | <0.682 |
| Thigh circumference difference (healthy - operated leg), cm | -0.06 (-0.10 to -0.02) | <0.006 | -0.10 (-0.14 to -0.06) | <0.001 | -0.08 (-0.11 to -0.05) | <0.001 |
| Knee joint pain during BFR-LLST, VAS-mm | -0.3 (-1.1 to 0.5) | <0.407 | -1.1 (-2.0 to -0.3) | <0.010 | -0.7 (-1.3 to -0.1) | <0.017 |
| Quadriceps muscle pain during BFR-LLST, VAS-mm | -0.2 (-1.48 to 1.15) | <0.801 | -0.7 (-1.9 to -0.5) | <0.241 | -0.4 (-1.3 to 0.4) | <0.330 |
| Rating of perceived exertion (8-20-point Borg Scale) during BFR-LLST, points | 0.1 (0.0 to 0.3) | <0.034 | 0.2 (0.1 to 0.3) | <0.001 | 0.2 (0.1 to 0.2) | <0.001 |
| Knee joint pain during usual care exercise, VAS-mm | -0.9 (-2.0 to 0.3) | <0.136 | -0.6 (-1.7 to 0.4) | <0.231 | -0.7 (-1.4 to 0.1) | <0.066 |
| Training load during BFR-LLST, kg | 0.30 (0.22 to 0.38) | <0.001 | 0.26 (0.21 to 0.31) | <0.001 | 0.28 (0.23 to 0.32) | <0.001 |

Abbreviations: CI; Confidence Interval. BFR-LLST; Blood flow restriction-low load strength training. VAS; Visual Analog Scale.

## S7 Table 4 Number of exercises performed during the group-based usual care exercise supervised program (15 sessions)

| Training modality | Exercise* | Number  Mean ± SD (min. to max.) | Total |
| --- | --- | --- | --- |
| Warm-up | Bike | 9 ± 2 (5 to 12) | 385 |
|  | Rowing machine | 0 ± 0 (0 to 0) | 0 |
| Warm-up in gym | Slides: Sliding walk | 8 ± 2 (3 to 11) | 347 |
|  | Forward lunge | 3 ± 2 (0 to 8) | 121 |
|  | Walking lunges with dumbbells | 0 ± 0 (0 to 2) | 8 |
|  | Mini Band Side Steps | 8 ± 2 (4 to 12) | 331 |
| Hip abduction strength | The clam w/ elastics | 4 ± 3 (0 to 11) | 176 |
|  | Side Plank on Knees with Leg Raises | 4 ± 2 (0 to 8) | 148 |
|  | Abduction of the hip with elastic band | 2 ± 2 (0 to 8) | 80 |
| Hamstring strength | ”Bridge” | 2 ± 2 (0 to 9) | 75 |
|  | Single Leg Glute Bridge, side view | 7 ± 2 (2 to 11) | 301 |
|  | Barbell Glute Bridge | 1 ± 1 (0 to 4) | 27 |
|  | Romanian Deadlift 2 | 0 ± 1 (0 to 6) | 16 |
|  | Single Leg Dumbbell Deadlift | 1 ± 2 (0 to 8) | 42 |
| Hip adductor strength | On your back "squeeze" of ball 1 | 3 ± 2 (0 to 11) | 129 |
|  | Side-lying single leg lift 2 | 5 ± 3 (0 to 10) | 218 |
|  | Standing inward leg pull w/elastic band 2 | 1 ± 1 (0 to 5) | 25 |
| Calf strength | Elevated Calf Raises | 1 ± 1 (0 to 6) | 58 |
|  | Elevated Calf Raises w/ dumbbells | 2 ± 1 (0 to 6) | 69 |
|  | Elevated Calf Raises – One-legged | 3 ± 2 (0 to 10) | 134 |
| Knee-extension strength | Cybex: Seated Leg Press 2 | 2 ± 2 (0 to 10) | 66 |
|  | Squat 1 | 3 ± 3 (0 to 11) | 123 |
|  | Goblet Squat | 0 ± (0 to 1) | 5 |
|  | Slides: Back Lunge | 3 ± 2 (0 to 9) | 142 |
|  | Slides: Back Lunge w/ dumbbells | 1 ± 2 (0 to 7) | 31 |
|  | Bulgarian Split Squat w/barbell | 0 ± 0 (0 to 2) | 2 |
| Balance and Stability | BOSU-ball: balance 1 | 0 ± 1 (0 to 2) | 15 |
|  | Balance board: single leg balance 1 | 0 ± 1 (0 to 2) | 13 |
|  | BOSU-ball: Balance 3 | 0 ± 0 (0 to 0) | 0 |
|  | BOSU-ball: Balance w/ weightball 2 | 0 ± 0 (0 to 0) | 0 |
| Stretching | The Extender | 0 ± 0 (0 to 1) | 1 |
|  | Buttocks 2 | 0 ± 0 (0 to 0) | 0 |
|  | Hip flexors 1 | 0 ± 1 (0 to 3) | 7 |
|  | Stretching of the front thigh 1 | 0 ± 1 (0 to 5) | 17 |
| Other treatments | Otreat (situps, manual mobilization, plank) | 7 ± (0 to 12) | 277 |

*Exercise names are corresponding to exercise names in S6 Usual care exercise after cartilage or meniscus repair in the knee joint - week 7 postoperatively

## S7 Table 5 Adverse events during the BFR-LLST added to usual care exercise intervention period

| Adverse events | Cartilage (n=21) | Meniscus (n=21) | Total (n=42) |
| --- | --- | --- | --- |
| Dizziness, events/patients, no | 25/9 | 27/10 | 52/19 |
| Quadriceps muscle pain at rest, events/patients, no | 0/0 | 1/0 | 1/1 |
| Knee joint pain at rest, events/patients, no | 3/2 | 2/2 | 5/4 |
| Numbness (transient), events/patients, no | 1/1 | 1/1 | 2/2 |
| Bruises, events/patients, no, events/patients, no | 2/2 | 2/1 | 4/3 |
| Cardiovascular complaints, events/patients, no | 0 | 0/0 | 0/0 |
| Deep Venous Thrombosis, events/patients, no | 0 | 0/0 | 0/0 |
| Other, events/patients, no  (Itching feelings in foot/lower leg, calf tightness, knee pain during exercise, discoloration, discomfort, slight nausea), events/patients, no | 73/19 | 73/19 | 146/38 |

## S7 Table 6 Average and overall change in knee joint pain, quadriceps muscle pain and perceived exertion from 1^st^ to 4^th^ set within the BFR-LLST session (15 sessions) for all patients (n=42)

|  | Change from set 1 | | Value per set^a^ |
| --- | --- | --- | --- |
| Outcome measure | Mean (95% CI) | p-value | Mean (95% CI) |
| Knee joint pain, VAS-mm |  |  |  |
| Set 1 |  |  | 5.9 (4.8 to 7.0) |
| Set 2 | -0.7 (-2.0 to 0.5) | <0.260 | 5.1 (4.2 to 6.1) |
| Set 3 | -0.6 (-1.9 to 0.6) | <0.328 | 5.1 (4.1 to 6.1) |
| Set 4 | -0.9 (-2.2 to 0.4) | <0.197 | 4.8 (3.7 to 5.9) |
| Type III test |  | <0.224 |  |
| Quadriceps muscle pain, VAS-mm |  |  |  |
| Set 1 |  |  | 11.1 (9.9 to 12.4) |
| Set 2 | 11.3 (9.3 to 13.3) | <0.0001 | 22.4 (20.7 to 24.2) |
| Set 3 | 21.3 (19.2 to 23.3) | <0.0001 | 32.2 (29.9 to 34.5) |
| Set 4 | 30.7 (28.6 to 32.7) | <0.0001 | 40.7 (37.8 to 43.6) |
| Type III test |  | <0.0001 |  |
| Rating of perceived exertion (8-20 point Borg Scale), points |  |  |  |
| Set 1 |  | <0.0001 | 10.8 (10.6 to 11.0) |
| Set 2 | 3.2 (3.0 to 3.4) | <0.0001 | 14.0 (13.8 to 14,1) |
| Set 3 | 5.5 (5.3 to 5.8) | <0.0001 | 16.3 (16.1 to 16.5) |
| Set 4 | 7.8 (7.6 to 8.1) | <0.0001 | 18.5 (18.3 to 18.7) |
| Type III test |  | <0.0001 |  |

BFR-LLST; Blood flow restriction - Low load strength training. CI; Confidence Interval. VAS; Visual Analog Scale. ^a^Value per set is an average of a specific set performed during 15 BFR-LLST supervised sessions for all patients (n=42).
